# Dynamic Interfacial Modulation in Pt@Ga Liquid Metal Systems

**DOI:** 10.1002/advs.202516511

**Published:** 2026-01-04

**Authors:** Zanyu Chen, Yixiao Zou, Wenda Chen, Chen Zhang, Jinfeng Zhang, Jia Ding, Xiaopeng Han, Wenbin Hu

**Affiliations:** ^1^ Tianjin Key Laboratory of Composite and Functional Materials, Key Laboratory of Advanced Ceramics and Machining Technology (Ministry of Education) School of Materials Science and Engineering State Key Laboratory of Precious Metal Functional Materials Tianjin University Tianjin 300350 China; ^2^ National‐Industry‐Education‐Platform of Energy Storage Tianjin University Tianjin 300350 China

**Keywords:** directional growth of platinum, hydrogen evolution reaction, liquid catalyst, metal‐support interaction, supported catalyst

## Abstract

Catalysts are essential in the transformation of chemical value chains, and traditional solid catalysts encounter challenges in structural flexibility, interfacial mass transport, and long‐term stability, especially in heterogeneous and electrochemical systems. Liquid metal‐based catalysts, particularly gallium (Ga), provide a dynamic platform with tunable physicochemical properties and favorable interfacial responsiveness. However, the electrochemical interfacial behavior and oxide regulation of Ga‐based liquid metal catalysts in alkaline electrolytes are still largely unexplored. In this work, Pt@Ga is selected as a model system and focused on the alkaline hydrogen evolution reaction (HER) to explore the potential of liquid metal‐based catalysts in the renewable hydrogen energy field. By precisely controlling the Ga surface oxide layer, the in situ formation of Pt wires is enabled with a high proportion of (200) crystal facets. This strategy overcomes the structural constraints of solid supports and enhances the electronic coupling between Pt and Ga. Experimental results from in situ analysis visualize Pt growth dynamics and reveal the synergistic interactions that accelerate charge and mass transport at the catalyst/electrolyte interface. This study presents a novel design for liquid metal‐based catalysts, where oxide‐regulated metal growth and dynamic interface evolution synergistically boost catalyst performance, offering a paradigm for next‐generation self‐adaptive systems.

## Graphical abstract

This work introduces a facile dissolution‐reconstruction approach to prepare the Pt@Ga liquid catalyst by dissolving Pt in molten Ga and then using electrochemical treatment to reconstruct Pt on the Ga support, which provides a new design strategy for liquid‐metal catalysts and enhances the understanding of oxide‐modulated segregation, offering new ideas for future research on multi‐functional supported catalyst systems.

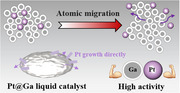

## 1 Introduction

Catalysts play a central role in the transformation of chemical value chains, with broad applications in energy production,^[^ ^1^ ^]^ storage,^[^ ^2^ ^]^ and environmental remediation.^[^ ^3^ ^]^ The catalytic performance, particularly in terms of activity and durability, is determined by multiple factors, including the material type, atomic arrangement at active sites, and resistance to long‐term degradation under harsh conditions.^[^ ^4^ ^]^ However, traditional solid catalysts are inherently constrained by the rigid lattice structures that bind their atoms, which restrict structural flexibility.^[^ ^5^ ^]^ As a result, these catalysts often exhibit poor adaptability in multi‐step reactions and relatively low stability.^[^ ^5^ ^]^ Furthermore, under severe operational conditions, catalyst deactivation and the need for regeneration lead to substantial economic and environmental costs.^[^ ^6^, ^7^ ^]^ A more critical challenge arises at the gas‐liquid‐solid interface, where solid catalysts often exhibit limited mass transport efficiency and poor dynamic response, which hinders the diffusion of reactants and products and may even cause passivation or structural degradation of active sites.^[^ ^8^ ^]^ These interface‐related issues are particularly pronounced in heterogeneous catalysis, representing a key bottleneck in the development of high‐performance catalytic material systems.^[^ ^9^ ^]^

In recent years, liquid metal‐based catalysts, especially gallium‐mediated systems, have emerged as promising alternatives for heterogeneous catalysis. Ga, with its low melting point (≈30 °C), eco‐friendliness, and distinctive physicochemical properties (including tunable Lewis acidity and high surface tension), offers a dynamic platform for catalyst design.^[^ ^10^, ^11^ ^]^ Compared to conventional solid‐supported catalysts, liquid Ga substrates maintain fluidity at the electrolyte/catalyst interface, offering low viscosity and high ionic/electronic conductivity, which synergistically enhance mass transport and interfacial charge transfer.^[^ ^12^, ^13^ ^]^ A notable advantage of liquid metal‐based catalysts lies in their liquid interface, which differs from the rigid interfaces of solid catalysts. The dynamic nature of the interface enables modulation of the local environment surrounding the active sites, thereby increasing the electron density at these sites and improving the utilization efficiency of active atoms.^[^ ^14^, ^15^ ^]^ More intriguingly, Ga is a dense, strongly interacting liquid that can directly dissolve neutral‐state metal atoms.^[^ ^16^ ^]^ This property allows for atomic‐level dispersion of solute atoms within the Ga matrix, providing a tunable environment for subsequent crystallization. As reported, solute metal atoms segregated from liquid alloys tend to form structures with specific symmetries or orientations, leading to materials with well‐defined crystal plane preferences.^[^ ^17^, ^18^ ^]^ The excellent fluid nature of Ga also enables it to act as a versatile solvent for various metal atoms, facilitating the synthesis of metal‐based catalysts with unique structures and compositions.^[^ ^19^ ^]^ In addition, the low viscosity endows liquid metals with self‐refreshing surface properties, ensuring efficient delivery of active sites and strong interfacial interactions with reactants.^[^ ^20^ ^]^ Given the unique advantages of liquid metals in terms of structural tunability, dynamic interface behavior, and adaptability, they hold great potential for application in future electrocatalysis,^[^ ^14^, ^21^, ^22^ ^]^ thermocatalysis,^[^ ^23^, ^24^, ^25^ ^]^ and photocatalysis.^[^ ^26^ ^]^ Moreover, although liquid metal‐based catalysts have been explored to some extent in electrocatalytic systems under neutral or mildly alkaline conditions, such as for CO_2_ reduction reaction and nitrogen reduction reaction.^[21,^ ^27^ ^].^ However, Ga is prone to oxidation upon exposure to air and aqueous environments, forming a surface oxide layer on Ga droplets that is difficult to remove.^[^ ^28^, ^29^ ^]^ As the application is extended to a strongly alkaline environment, the electrochemical interfacial behavior and oxide layer regulation of liquid metal‐based catalysts remain largely unexplored.

To further explore the potential of liquid metal‐based catalysts in the renewable hydrogen energy field, we selected Pt@Ga as a model system and focused on the alkaline hydrogen evolution reaction, as Pt is the most widely used benchmark catalyst due to its optimal hydrogen adsorption energy, high exchange current density, and low overpotential.^[^ ^30^, ^31^ ^]^ However, conventional carbon‐supported Pt catalyst still suffers from weak metal‐support interactions, structural instability, and poor corrosion resistance during long‐term operation.^[^ ^32^, ^33^, ^34^, ^35^ ^]^ To address these issues, we leveraged the fluidity and reconfigurability of liquid metal Ga to achieve dynamic interfacial regulation and enhanced mass transport at the solid‐liquid‐gas interface. In this work, we propose a dissolution‐reconstruction strategy for preparing liquid metal‐based catalysts, which enables the in situ generation of Pt wires with a high proportion of (200) crystal facets on the Ga support. The existence of the Ga surface oxide layer in alkaline electrolytes plays a key role in regulating the growth behavior of Pt. Our work combines in situ optical microscopy, Electrochemical Impedance Spectroscopy (EIS), and Raman spectroscopy to directly visualize the Pt segregation dynamics and their correlation with HER performance during electrochemical reconstruction. Additionally, the synergistic interaction between Pt and liquid Ga in the HER process accelerates charge/mass transport at the catalyst/electrolyte interface. Ga‐Pt electron transfer mechanism results in a negatively charged Pt surface, which induces the formation of a locally acidic environment on the Pt surface due to the electrostatic attraction of H_3_O^+^ ions, significantly enhancing water dissociation kinetics during the HER process. This work demonstrates a promising approach to utilizing liquid metal as a dynamic and reconfigurable catalyst support, and provides fundamental insights into oxide‐regulated metal growth and interfacial evolution in electrochemical systems. It offers a fresh perspective for exploring fundamental metal‐liquid interactions and designing next‐generation electrocatalytic material systems.

## 2 Results and Discussion

**Figure 1:**
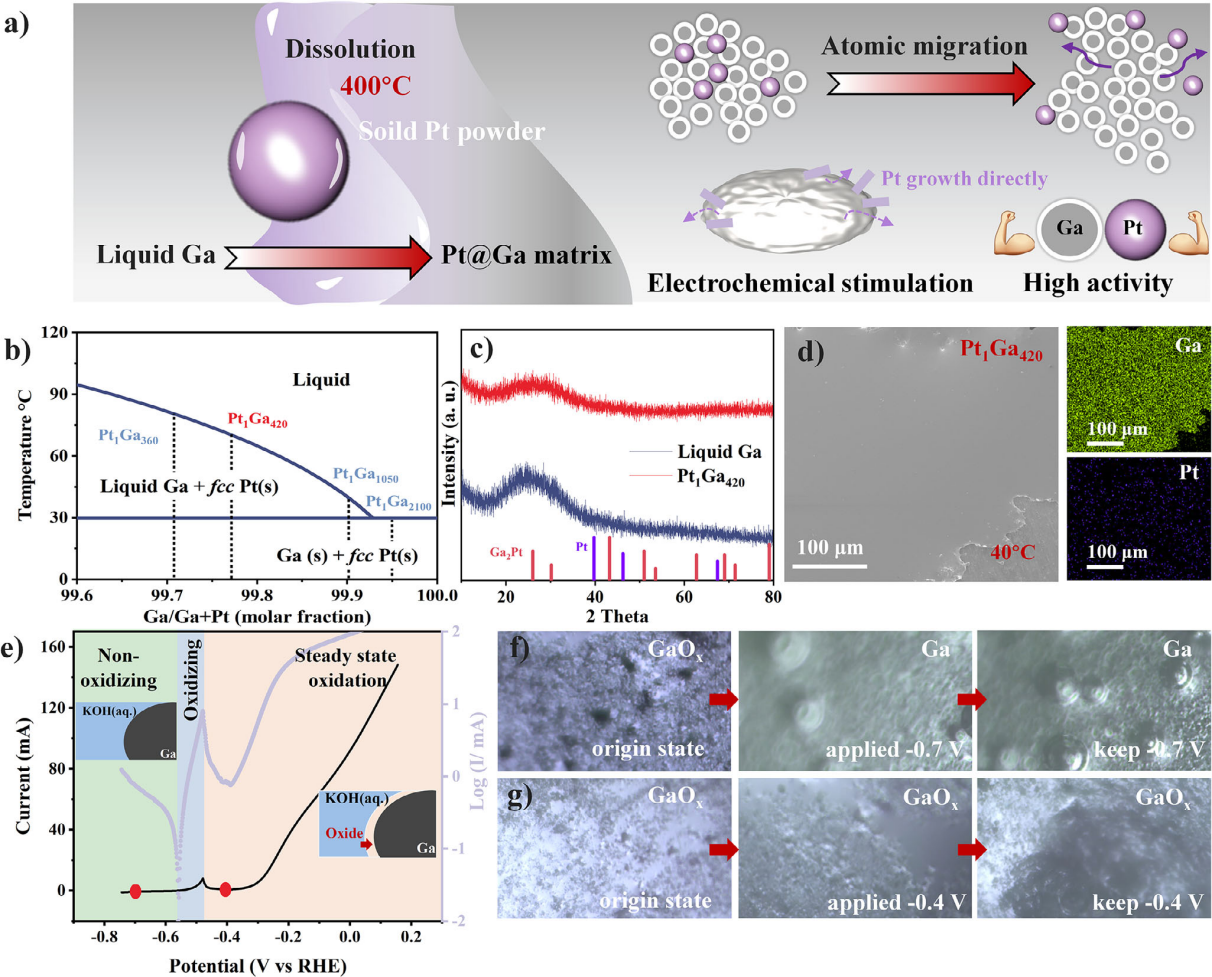
a) Synthetic schematic diagram of Pt@Ga matrix. b) Phase diagram of Pt@Ga system in the temperature range of 0–120 °C. c) The XRD pattern and d) the corresponding SEM and the EDX mapping images of Pt_1_Ga_420_. e) The electrochemical behavior of Pure Ga. The in situ optical microscope image of Ga_1_Pt_420_ with activating behavior in different potentials of f) −0.7 V and g) −0.4 V.

The Pt@Ga matrix was prepared via a dissolution‐reconstruction method, as illustrated in **Figure** **1**a. Solid Pt powder was dissolved in molten Ga at ≈400 °C to form a stable liquid alloy, which was then cooled for HER evaluation. High‐temperature dissolution replaces conventional physical stirring, enabling atomic‐scale dispersion of Pt atoms within the Ga matrix.^[^ ^36^ ^]^ Under electrochemical stimulation, these Pt atoms migrate and reorganize from the bulk Ga to surface domains. As shown in Figure 1b, the phase diagram analysis shows that Pt atoms remain atomically dispersed in the Ga matrix below the solubility limit at ≈400 °C. Upon cooling, Pt atoms undergo nucleation and local rearrangement, resulting in the coexistence of liquid Ga and solid Pt.^[^ ^17^ ^]^ Upon cooling to 29.8 °C, the liquid alloy undergoes complete solidification, forming solid Ga and solid Pt. As shown in Figure S1 (Supporting Information), Pt powder and a liquid Ga droplet were placed in a graphite boat within an Ar atmosphere and then heated for 5 h. After heating, the black Pt powder attached to the surface of the liquid metal dissolves and disappears. Meanwhile, the surface of the liquid metal retains a distinct metallic feature. The X‐ray diffraction (XRD) pattern in Figure 1c and Figure S2 (Supporting Information) reveals that no significant Pt crystalline phase diffraction peaks emerge across samples with varying Pt/Ga ratios (Pt_1_Ga_360_, Pt_1_Ga_1050_, Pt_1_Ga_2100_), indicating that Pt atoms maintain an amorphous state within the Ga matrix. However, no Pt crystal phase peaks were detected in the high Pt content sample Pt_1_Ga_360_, but its peak shape was clearly different from the amorphous structure of pure Ga. This difference is due to partial Pt precipitation and local alloying within the Ga matrix. The scanning electron microscope (SEM) images of Pt_1_Ga_420_ sample in Figure 1d and Figure S3 (Supporting Information) reveal that the obtained Pt@Ga alloy remains smooth and shows no Pt particle aggregation, while energy dispersive X‐ray spectroscopy (EDX) mapping confirms uniform Pt/Ga distribution. Furthermore, the high‐resolution transmission electron microscopy (HRTEM) images in Figures S4 and S5a–c (Supporting Information) show that the small‐sized nanocrystals have clear lattice fringes with an interplanar spacing of ≈0.19 nm, which is inferred to be the (200) crystal plane of Pt. In addition, EDX mapping analysis shown in Figures S5d–f (Supporting Information) further confirms that the composition of these small grains is primarily Pt element. These results demonstrate that Pt nanocrystals are dispersed within the Ga matrix, suggesting that recrystallization occurred during the cooling process, in consistent with phase diagram predictions. To determine the optimal electrochemical activation potential, linear sweep voltammetry (LSV) was performed on pure Ga droplets in 1 M KOH. The current‐potential curve (Figure 1e) identifies three distinct electrochemical regions: 1) surface oxidation initiates at E > ‐0.48 V (vs RHE), forming a thin Ga oxide layer; (2) oxide dissolution occurs between −0.48 and −0.55 V; 3) rapid dissolution of the surface oxide layer proceeds at E < −0.55 V.^[^ ^37^ ^]^ Control experiments at −0.7 and −0.4 V (vs RHE) were conducted to assess oxide layer effects on Pt@Ga. As shown in Figure 1f,g, the in situ optical microscopy shows that Pt_1_Ga_420_ behaves similarly to pure Ga. At −0.7 V, the oxide layer dissolves rapidly, accompanied by immediate hydrogen bubble generation on the exposed Ga surface. However, at −0.4 V, the stable oxide layer remains intact, and hydrogen bubbles are observed to form on the wrinkled oxide layer. Moreover, the lower current density at −0.4 V results in slower bubble growth.^[^ ^38^ ^]^

**Figure 2:**
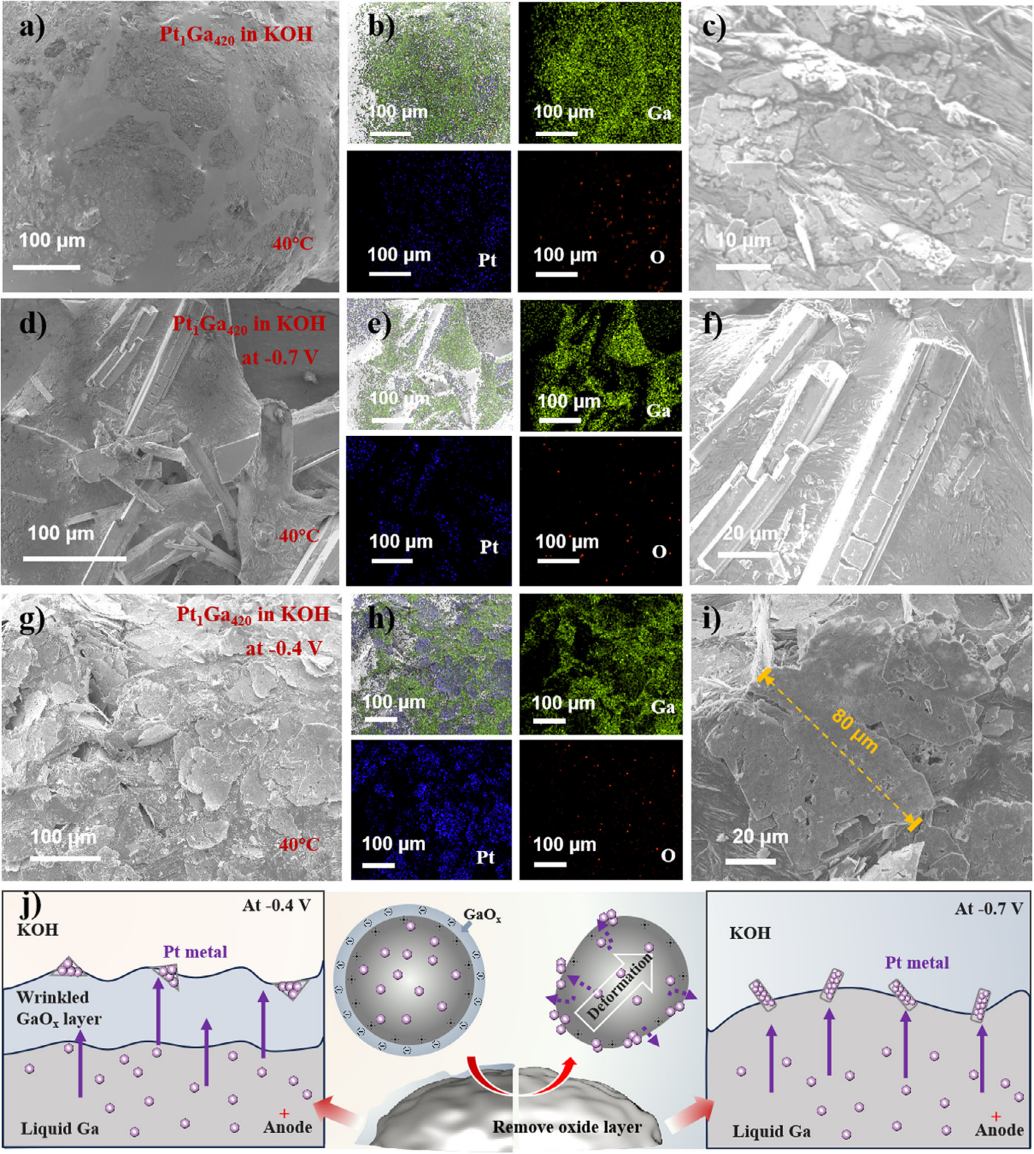
The SEM images and corresponding EDX‐mapping characterization of liquid metal‐based catalysts after different voltage regulation. a–c) The Pt_1_Ga_420_ immersed in KOH without voltage application. d‐f) The Pt_1_Ga_420_ activated in KOH at the potential of −0.7 V for 6000 s. g–i) The Pt_1_Ga_420_ activated in KOH at the potential of −0.4 V for 6000 s. j) The mechanism diagram of the Pt inward‐to‐outward growth phenomenon.

An open three‐electrode system was built at 40 °C, where the Pt@Ga loaded on the groove with an internal copper wire served as the working electrode, a carbon rod as the counter electrode, and Hg/HgO as the reference electrode. Voltage application induced substantial gas evolution at the catalyst surface (Figure S6, Supporting Information), while SEM analysis after 6000 s of stimulation revealed distinct morphologies (**Figure** **2**a‐i). When the Pt@Ga mixture was immersed in KOH without voltage application, a few Pt plates (≈10 µm) formed on the liquid‐metal surface (Figure 2a–c). This phenomenon is attributed to the slow release of Ga(OH)_4_^−^ from the liquid‐metal droplet in the alkaline solution, and this process generates a negatively charged Ga surface, fostering a double electric layer and driving slow Pt nanoparticle deposition through internal charge redistribution.^[^ ^39^ ^]^ When activated at −0.7 V (vs RHE) without an oxide layer, Pt particles self‐assembled into well‐ordered linear structures (Pt@Ga‐wire) (Figure 2d–f). The elemental mapping images in Figure 2e reveal distinct stratification between Ga and Pt, with no detectable Ga on the Pt wires. In contrast, at −0.4 V with an oxide layer present, Pt was deposited as thick and stacked plates (≈80 µm), which are far larger than those from spontaneous growth (Figure 2g–i). Further experiments at intermediate potentials (−0.5, −0.6, −0.8 V vs RHE) revealed a gradient in oxide layer modulation. At −0.5 V, partial oxide formation led to short nanosheet‐like Pt deposits (Figure S7a, Supporting Information). At −0.6 V, partial oxide dissolution resulted in coexisting plate‐like and wire‐like Pt structures (Figure S7b, Supporting Information). At −0.8 V, the oxide layer quickly breaks down, causing fast Pt precipitation that forms ultralong Pt wires (>300 µm), and this breakdown also leads to Ga surface etching and small holes on the substrate (Figure S7c, Supporting Information). Notably, when Pt powder was physically mixed with Ga without high‐temperature dissolution (Pt mixed in Ga), voltage application produced coarse Pt aggregates (≈30 µm) on the surface, highlighting the necessity of thermal dissolution for atomic‐level dispersion (Figure S8, Supporting Information). The results demonstrate that the Ga surface oxide layer plays a key role in regulating the growth behavior of Pt. As illustrated in Figure 2j, when an oxide layer is present, the catalyst‐electrolyte interface can be divided into metal, metal oxide, and electrolyte. The deposition of Pt is controlled by the triple interaction of liquid metal, oxide layer, and electrolyte, leading to the formation of thick, irregular plates.^[^ ^17^, ^40^ ^]^ Conversely, under more negative potential (−0.7 V), the oxide layer dissolves, leaving only the metal‐electrolyte interface. Here, the high interfacial tension of Ga and significant surface energy changes under voltage direct reorganization, enabling ordered linear Pt structures.^[^ ^41^ ^]^

**Figure 3:**
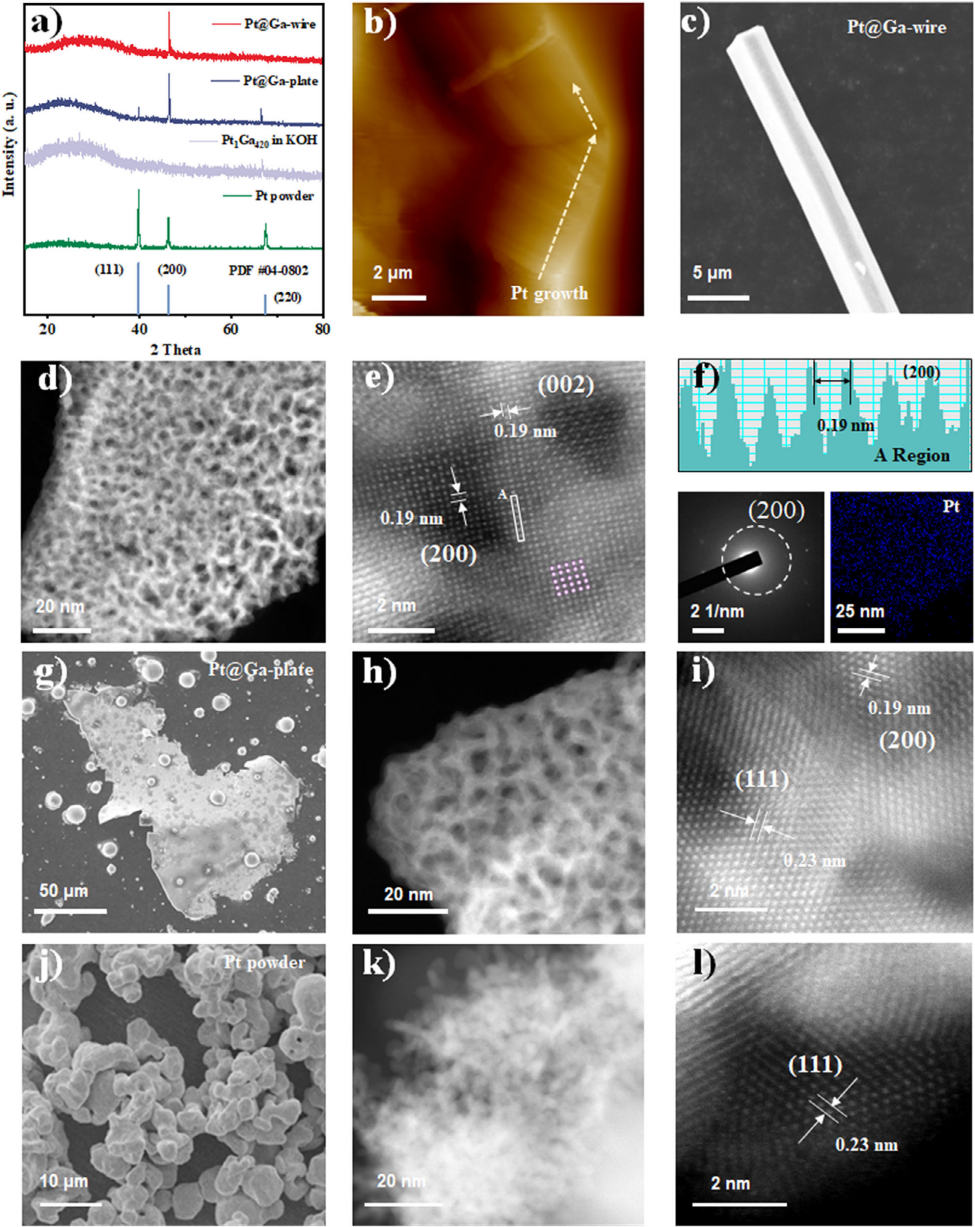
a) The XRD patterns and b) the AFM images of Pt@Ga‐wire. c) The SEM image, b‐e) the HAADF‐STEM images, f) the linear scanning of the A region marked in e, the SAED pattern, and elemental mapping of Pt@Ga‐wire. g) The SEM image, h–i) the HAADF‐STEM images of Pt@Ga‐plate. j) The SEM image, k‐l) the HAADF‐STEM images of Pt powder (raw material).

The crystal phase of Pt under different growth conditions was characterized via XRD in **Figure** **3**a. The original Pt powder exhibits a face‐centered cubic (fcc) structure (PDF#04‐0802), with the strongest peak at 2θ = 39.8° corresponding to the Pt (111) plane.^[^ ^42^ ^]^ In contrast, no Pt diffraction peaks were detected for the Pt_1_Ga_420_ sample after alkaline soaking (Pt_1_Ga_420_ in KOH), suggesting the low surface aggregation of segregated Pt in the Ga surface. However, the Pt@Ga‐wire and Pt@Ga‐plate samples displayed distinct crystallographic orientations relative to the Pt powder. The Pt@Ga‐plate sample shows an enhanced intensity for the Pt (200) plane compared to the (111) and (220) planes, while Pt@Ga‐wire exhibits only the (200) diffraction signal. This absence of other Pt diffraction peaks indicates preferential (200) plane growth during segregation from high surface tension liquid Ga. Atomic force microscopy (AFM) further analyzed the surface topography of these structures. As depicted in Figure 3b and Figure S9 (Supporting Information), the AFM characterization of Pt@Ga‐wire reveals a distinctive linear growth pattern. This morphology might arise from a surface energy‐driven directional crystallization process, where Pt atoms preferentially align along low‐energy crystallographic directions during segregation from the liquid metal substrate.^[^ ^18^ ^]^ For the Pt@Ga‐plate, the AFM reveals pronounced surface undulations with an average roughness (R_a_) of 30.7 nm, attributed to its polycrystalline interpenetrating network. To further characterize the internal structure, the excess polycrystalline interpenetrating network was studied. To further characterize the internal structure, the excess Ga substrate was etched using hydrochloric acid. As shown in Figure 3c, the Pt@Ga‐wire still retains a linear morphology after Ga removal. The HAADF‐STEM image (Figure 3d) reveals that the synthesized Pt wires exhibit a unique 3D continuous interpenetrating network structure, featuring abundant nanoscale channels (2–5 nm), which would enhance active site accessibility and mass transport for improved catalytic performance.^[^ ^43^, ^44^ ^]^ This hierarchical structure might originate from simultaneous outward Pt metal growth and vigorous H_2_ bubble evolution during migration and growth. The high density of H_2_ bubbles acts as a dynamic template, interrupting continuous Pt through inward‐to‐outward growth and forming a hierarchical wire morphology.^[^ ^45^ ^]^ The dominant (200) crystal plane (d‐spacing = 0.19 nm) in the porous Pt matches the XRD results, and line‐scan analysis confirms abundant lattice vacancies within the (200) plane (Figure 3e). Selected‐area electron diffraction (SAED) and elemental mapping analysis of the Pt wire confirm that the wire‐like Pt is a pure Pt phase, with no Ga alloying detected (Figure 3f). Moreover, similar structural insights emerge for the Pt@Ga‐plate. The SEM and High Angle Annular Dark Field‐Scanning Transmission Electron Microscopy (HAADF‐STEM) images in Figure 3g,h reveal that the Pt@Ga‐plate similarly exhibits a 3D interpenetrating network structure of plate, indicating the architecture originates from the unique segregation pathway inherent to the liquid Ga substrate. This structure contains mixed polycrystalline facets, including (111) and (200) planes (Figure 3i). Notably, both wire and plate morphologies starkly differ from the original Pt powder, which formed large agglomerates through particle stacking with predominant (111) orientation (Figure 3j–l).

The regulation of Pt growth by initial Pt content and voltage stimulation patterns was systematically studied. As shown in Figure S10 (Supporting Information), under constant potential (−0.7 V) and temperature (40 °C), no micrometer‐scale Pt wires were observed in Pt_1_Ga_2100_ due to insufficient Pt atomic concentration. Conversely, Pt_1_Ga_1050_ consistently formed oriented Pt wires, with wire diameter showing a positive correlation with Pt content (≈20 µm) (Figure S11, Supporting Information). For Pt_1_Ga_360_, the system exhibited coexisting Pt wires and plate‐like Pt structures (Figure S12, Supporting Information). Voltage stimulation patterns also strongly influenced Pt precipitation in Pt_1_Ga_420_. When continuous stimulation (6000 s duration) was replaced with intermittent protocols (50, 20, or 10 s intervals), the phase composition of the precipitates changed obviously (Figure S13a, Supporting Information). As shown in Figure S13b (Supporting Information), shorter intervals (20 and 10 s) lead to the formation of mixed Pt particles with reduced crystallographic orientation, coexisting with the alloy phases Ga_7_Pt_3_ and Ga_2.7_Pt_5.3_. In contrast, when the interval is extended to ≈50 s, the system returns to forming well‐oriented Pt wires predominantly aligned along the (200) crystal plane, with no detectable alloy phases. The SEM images in Figure S14 (Supporting Information) further confirm that in the short stimulation mode (10 and 20 s intervals), Pt does not have sufficient time to grow out from the interior of the Ga matrix. As a result, only a limited number of short Pt lines are formed, and a significant amount of Pt remains encapsulated within the Ga, leading to the formation of Pt‐Ga compounds. By contrast, the intermittently stimulated Pt wires with a 50 s interval maintain a linear morphology (Figure S15a, Supporting Information), as evidenced by elemental mapping and line‐scan analysis (Figures S15b,c, Supporting Information), which confirm their pure Pt composition without Ga incorporation.

**Figure 4:**
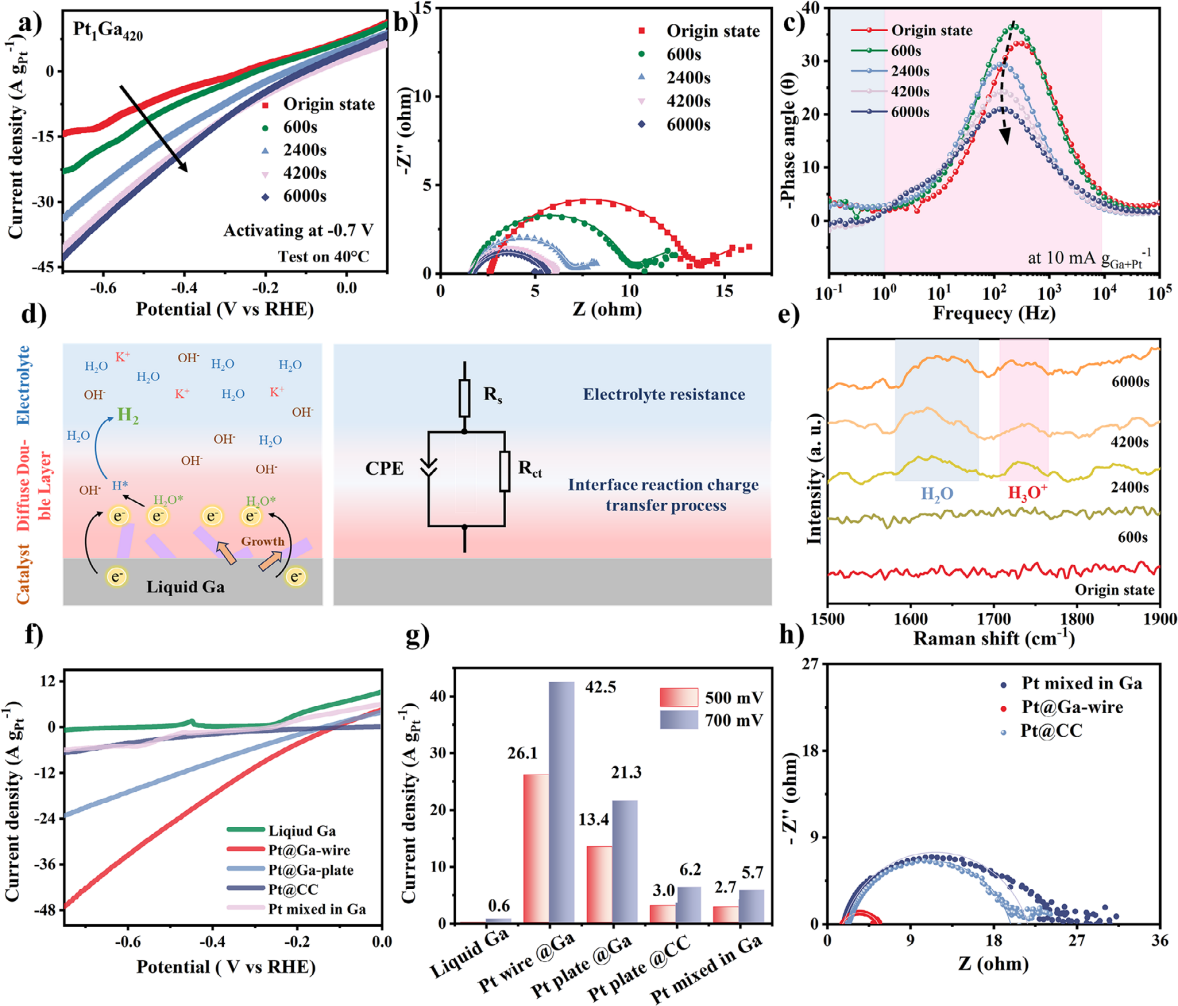
a) The HER activity of the Pt@Ga mixture in the electrochemical activation process. In situ EIS measure of b) Nyquist plot, c) Bode phase plots, and d) the corresponding equivalent circuit model of Pt@Ga mixture. e) In situ Raman spectrum of Pt@Ga mixture in the electrochemical activation process. f) The HER performance and g) their comparison of Liquid Ga, Pt@Ga‐wire, Pt@Ga‐plate, Pt mixed in Ga, and Pt@CC. h) The Nyquist plot comparison of Pt@Ga‐wire, Pt mixed in Ga, and Pt@CC.

Based on the structural analysis, we tested the catalytic activation of Pt_1_Ga_420_ under a constant potential of −0.7 V. The HER current density exhibits a significant increased values with prolonged activation time (**Figure** **4**a). The nearly constant current observed between 4200 and 6000 s implies that the reconstructed Pt‐Ga interface has achieved structural stability. Inductively coupled plasma‐Mass Spectrometry (ICP‐MS) results confirmed no Pt dissolution into the electrolyte after activation, proving the Pt wires adhered strongly to the Ga substrate. Meanwhile, this activation process facilitates the migration of active Pt atoms from the interior of the Ga matrix to the surface interface, resulting in the exposure of more active sites, which endow great electrocatalytic performance (Figure S16, Supporting Information).^[^ ^46^ ^]^ Further mechanistic insights were gained through in situ EIS during activation (Figure 4b,c). Nyquist plots were fitted using a simplified Randles circuit as the equivalent circuit (Figure 4d), including the constant phase element (CPE), R_s_ (electrolyte resistance), and R_ct_ (interfacial charge‐transfer resistance). The R_ct_ value decreased progressively during activation, indicating Pt wire growth improved electron conduction in the catalyst bulk and accelerated surface reactions (Table S1, Supporting Information).^[^ ^47^, ^48^, ^49^ ^]^ This conclusion is reinforced by the reduction in phase angle within the mid‐frequency region, which corresponds directly to facilitated charge transfer at the electrode/electrolyte interface (Figure 4c). Moreover, the Pt_1_Ga_420_ under a constant potential of −0.4 V also exhibits a similar activation trend. As shown in Figure S17a (Supporting Information), the current density increases gradually with the activation time. Notably, after the activation process, the Pt_1_Ga_420_ sample under a constant −0.7 V shows a lower resistance compared to that under −0.4 V, suggesting a more effective catalyst activation at the lower potential (Figure S17b, Supporting Information). In situ Raman spectroscopy was employed to monitor interfacial structural changes (Figure 4e). After activation, a sharp H─O─H bending vibration peak at 1630 cm^−1^ appeared, indicating enhanced surface hydrophilicity due to the exposure of Pt wires, which facilitates water adsorption at active sites.^[^ ^50^ ^]^ Simultaneously, a distinct peak at 1750 cm^−1^ emerged, which is attributed to H_3_O^+^ intermediate species.^[^ ^51^ ^]^ For comparison, LSV curves of Liquid Ga, Pt@Ga‐wire, Pt@Ga‐plate, Pt@CC, and Pt mixed in Ga were measured in the HER region (Figure 4f). Pt@Ga catalysts derived from liquid metal demonstrated superior HER activity, with Pt@Ga‐wire achieving current densities of 26.1 and 42.5 A g_Pt_^−1^ at 500 and 700 mV, respectively, outperforming all other materials (Figure 4g). Moreover, the hydrogen evolution currents of Pt@Ga materials with varying Pt/Ga ratios increase during the activation process (Figure S18, Supporting Information). While the HER performance is positively correlated with Pt content, an excessively high Pt loading could lead to a reduction in mass‐normalized current density (Figure S19, Supporting Information). The effect of temperature on the activation of HER performance was also investigated (Figure S20, Supporting Information). No significant activation behavior caused by Pt migration to the surface was observed at either 20 or 80 °C, as the material forms a solid mixture at ≈20 °C and remains a homogeneous liquid at ≈80 °C (Figure 1b). It is important to note that the increase in current density with rising temperature under the same overpotential cannot be directly attributed to the intrinsic activity of the Pt@Ga material, since temperature can influence the hydrogen production process via water splitting.^[^ ^52^, ^53^ ^]^ All Ga‐containing liquid metal catalysts exhibit a positive charge response at low potentials, which is attributed to the partial oxidation of Ga droplets in alkaline solutions.^[^ ^36^, ^37^, ^54^, ^55^ ^]^ As shown in Figure S21 and Table S3 (Supporting Information), the Pt@Ga‐wire catalyst exhibits the lower overpotential of 149 mV at a current density of 10 mA cm^−2^, which is better than that of Pt@Ga‐plate (191 mV), Pt mixed in Ga (479 mV), and Pt@CC (340 mV). The EIS results show Pt@CC and Pt mixed in Ga had similar R_ct_ values, indicating that physically mixed Ga merely functions as a conductive support (Figure 4h; Table S2, Supporting Information). In contrast, Pt@Ga‐wire exhibits ultra‐low R_ct_, confirming rapid charge transfer through strong electronic coupling between Pt wires and Ga substrate.^[^ ^13^ ^]^ These findings demonstrate that electrochemical activation in liquid metals constructs a synergistic Pt‐Ga interface, delivering HER performance superior to conventional solid Pt‐based catalysts. Moreover, during the activation process, Ga dissolves to a certain extent. Upon completion of the activation (6000 s), the Ga content is ≈51.26 mg L^−1^, indicating a certain degree of dissolution. However, as the voltage application time is extended, the change in Ga content becomes relatively insignificant (Table S4, Supporting Information). These results suggest that the activation process promotes the formation of a Pt‐coated layer on the surface of liquid Ga, which effectively delays the dissolution of internal Ga and reduces its overall dissolution in the system.

**Figure 5:**
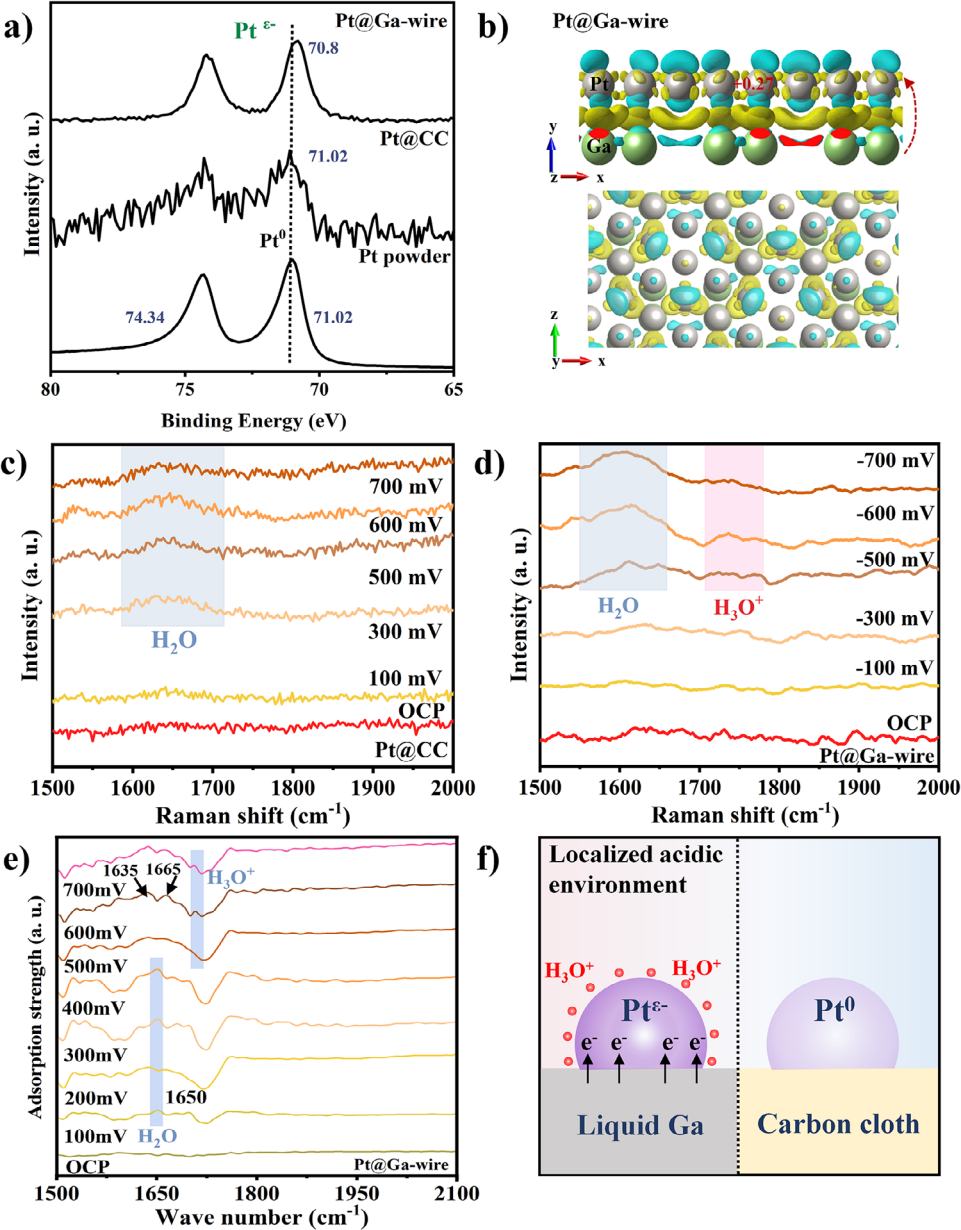
a) The 4f XPS spectra of Pt@Ga‐wire, Pt@Ga‐plate, Pt powder and Pt@CC. b) Surface charge density differences of Pt@Ga and Pt@CC model. The yellow and blue colors in the plot represent the accumulation and dissipation of the charges, respectively. In situ Raman spectra of c) Pt@CC and d) Pt@Ga‐wire. e) In situ ATR‐SEIRAS spectra of Pt@Ga‐wire. f) Schematic illustration of the localized environment over Pt@Ga and Pt@CC.

To further explore the electronic interaction between liquid Ga and precipitated Pt, the electronic properties of Pt@Ga‐wire were compared with Pt powder and Pt@CC, using X‐ray photoelectron spectroscopy (XPS) (**Figure** **5**a). The Pt 4f XPS spectra show that the two spin‐orbit doublets of Pt powder and Pt@CC could be divided into 71.02 and 74.34 eV, which are assigned to metallic Pt (0).^[^ ^56^ ^]^ The peak of Pt@Ga‐wire shifts negatively by ≈0.27 eV relative to that of Pt powder, directly proving that the Pt active sites on the Ga support show an electron‐rich characteristic property.^[^ ^57^, ^58^ ^]^ Conversely, Ga 3d spectra showed a positive shift, consistent with electron depletion in Ga, which may suggest that electron transfer occurs reversely from the Ga support to Pt wire (Figure 5b).^[^ ^59^ ^]^ Meanwhile, the XPS analysis of the 3d and 2p orbitals of Ga was also conducted. As shown in Figure S22 (Supporting Information), two distinct peaks are observed in the Ga 2p_3/2_ spectrum. The peak at 1118.1 eV is attributed to gallium oxide, likely caused by minor oxidation under the testing conditions, and the peak at 1115.5 eV corresponds to metallic Ga (0). Compared to pure Ga, the Ga 2p_3/2_ peak in the Pt@Ga‐wire catalyst shifts to a higher binding energy, indicating a stronger electron transfer effect between the Ga support and the Pt wire. A further comparison of the high‐resolution XPS spectra of Ga 3d for pure Ga and Pt@Ga‐wire shows this electron transfer effect to be even more pronounced. The peak at ≈17.99 eV corresponds to metallic Ga (0). This was further supported by the density functional theory (DFT) derived electron density and Bader charge analysis. Pt atoms on the Ga substrate exhibit electron‐rich features, as shown in blue isosurfaces. Notably, the charge transfer direction differs between Pt and the substrate. For Pt@Ga‐wire, ≈0.27 electrons were transferred from Ga to Pt, enhancing the electron‐rich character of Pt. This strategy of electronic transfer between liquid metals and active metals has also been reported in other liquid metal‐based systems.^[^ ^60^, ^61^ ^]^ Moreover, to further investigate the impact of electronic modulation on the catalyst during the electrocatalytic process, we employed in situ Raman spectroscopy and attenuated total reflectance Fourier‐transform infrared spectroscopy (ATR‐SEIRAS) to study the evolution of surface reactive intermediates on Pt@Ga wires and Pt@CC during the reaction. As shown in Figure 5c,d, all catalysts show adsorbed water bending vibrations at ≈1630 cm^−1^ under low to high overpotentials. When the potential exceeds ‐500 mV, Pt@Ga‐wire shows a distinct H_3_O^+^ signal at 1750 cm^−1^, whereas Pt@CC shows no such feature. As shown in Figure 5e and Figures S23–S25 (Supporting Information), the ‐OH stretching peak of Pt@Ga‐wire at 3218 cm^−1^ displayed a blue shift and intensity enhancement compared to Pt@CC at 3200 cm^−1^ and Pt‐wire@CC at 3210 cm^−1^, indicating the liquid Ga substrate markedly enhances interfacial water transport kinetics and accelerates dissociation.^[^ ^62^ ^]^ Upon exceeding −500 mV, the above‐mentioned characteristic absorption peak of *H_2_O occurs splitting as 1635 and 1665 cm^−1^, suggesting Ga participated in the water adsorption process. Meanwhile, two new characteristic peaks at 1700 and 2033 cm^−1^ were detected, representing the vibrational modes of hydronium ions (H_3_O^+^), respectively.^[^ ^63^, ^64^, ^65^ ^]^ The HER kinetics in alkaline media are significantly slower than those in acidic media due to the additional water dissociation step required for the supply of adsorbed hydrogen (*H), which typically results in a 2‐3 order of magnitude lower activity.^[^ ^66^, ^67^, ^68^ ^]^ As illustrated in Figure 5d,e, H_3_O^+^ accumulates at a high concentration on the surface of the Pt@Ga‐wire catalyst, creating a localized acidic environment and thereby improving the efficiency of water dissociation.^[^ ^69^ ^]^ In contrast, no H_3_O^+^ signals were observed in Ga‐free materials such as Pt@CC and Pt‐wire@CC samples. Overall, the fast charge transfer between Pt and Ga in the Pt@Ga‐wire catalyst leads to a negatively charged Pt surface, which effectively attracts H_3_O^+^ through electrostatic interactions, thereby enhancing water‐splitting performance at high current densities (Figure 5f).^[^ ^70^ ^]^ Furthermore, the liquid Ga metal substrate endows the catalyst with unique self‐healing capability. After washing the deactivated Pt@Ga material and transferring it to the glovebox for reheating, the resulting material exhibited a smooth surface, and no distinct Pt characteristic diffraction peaks were observed in the XRD analysis (Figure S26, Supporting Information). This indicates that the precipitated Pt has redissolved into the Ga matrix, restoring its structural homogeneity. When the repaired electrode is subjected to the same potential again, it still undergoes a reactivation process. After applying −0.7 V for 6000 s, the highly oriented Pt wires reprecipitate, and the electrochemical performance is restored to its high catalytic activity (Figure S27, Supporting Information).

## 3 Conclusion

This work presents a strategy for designing Pt@Ga liquid catalysts via a dissolution and reconstruction approach. The catalyst was prepared by dissolving Pt in molten Ga, followed by voltage‐controlled restructuring to form Pt wires with preferential (200) crystal orientation. Due to the electron transfer from Ga to Pt during the restructuring process, the Pt surface becomes negatively charged. Under high potentials, this specific negative charge of Pt enhances the electrostatic attraction of H_3_O^+^ ions, creating a localized acidic environment that accelerates water dissociation kinetics. Moreover, compared to conventional solid‐supported catalysts, the Pt@Ga system shows dynamic reversibility through Ga‐mediated regeneration, where thermal redissolution and reactivation restore its structure and performance. This study should shed light on the controllable synthesis of liquid metal‐based catalysts and the understanding of the electrocatalytic behavior in aqueous solutions for the electrically driven catalytic research field.

## 4 Experimental Section

### Chemical Reagent

The Pt (99.99%, purity) powder was purchased from Heowns Biochem LLC (Tianjin, China). Metal Ga was obtained from Macklin Biochemical Technology Co., Ltd (Shanghai, China). Potassium hydroxide (KOH) and hydrochloric acid (HCl) were purchased from Sinopharm Chemical Reagent. The carbon cloth was obtained from CeTech Co., Ltd. Deionized water was prepared from a Smart‐N System (Heal Force) in all the experiments.

### Preparation of Pt@Ga Electrodes

Pt@Ga binary liquid alloys (Pt_1_Ga_x_) were synthesized by precisely weighing Pt powder and Ga droplets in an argon‐filled glovebox, followed by loading them into a graphite boat (custom‐made by Hefei In situ Technology Co., LTD.). The mixture was melted at 400 °C on a hot plate for 5 h to ensure homogeneity. After cooling to 90 °C, the liquid alloy was collected, sealed in an argon atmosphere, and stored in an oven kept at 90 °C. Based on the atomic ratios of Pt: Ga (1:420, 1:360, 1:1050, and 1:2100), the prepared samples were named Pt_1_Ga_420_, Pt_1_Ga_360_, Pt_1_Ga_1050_, and Pt_1_Ga_2100_, respectively.

### Activation of the Pt@Ga Catalyst

The electrodes were activated using a homemade electrochemical system: 1 g Pt_1_Ga_x_ sample served as the working electrode, a saturated calomel electrode as the reference electrode, and a graphite rod as the counter electrode. A concave platform embedded with a Cu wire established a connection to the flowing liquid metal. During activation, the system was operated under controlled potential conditions to regulate the Ga surface oxide layer, enabling the in situ reconstruction of Pt structures. The electrochemical behavior was monitored via linear sweep voltammetry (LSV) and cyclic voltammetry (CV) in 1 M KOH solution. The activation process at −0.7 V (vs RHE) yielded highly ordered Pt wires with (200) crystal plane orientation, named as Pt@Ga‐wire, while activation at −0.4 V resulted in irregular Pt plate‐like structures named as Pt@Ga‐plate.

### Material Characterization

The X‐ray diffraction (XRD) patterns were conducted using a Rigaku SmartLab X‐ray diffractometer (Cu Kα radiation, 40 kV, 40 mA) to determine crystal phases of Pt@Ga catalysts The Pt@Ga materials were coated on clear Cu foil, and then performed on a JEOL tungsten hairpin filament scanning electron microscope and the field emission environmental scanning electron microscope (Thermo Fisher Scientific Quattro S) to visualize morphology of surface and confirm elemental distribution. The chemical states of elements in Pt@Ga materials were confirmed by X‐ray photoelectron spectroscopy (XPS) for Thermo Fisher Escalab 250X. Moreover, the content of Pt leaching in post‐activation electrolyte was measured by inductively coupled plasma mass spectrometry (ICP‐MS) (Agilent 7900). Because liquid Ga was not resistant to strong electron irradiation and was prone to volatilization. Therefore, the tested Pt@Ga material was added to an adequate amount of 1 M HCl and left at 60 °C for 12 h. Then, it was washed with water, filtered, the pH was adjusted to neutral, and dried. The excess Ga element was washed away, and the gray Pt product was obtained. The structures and crystallographic orientation of Pt products were tested by HRTEM and HAADF‐STEM (JEOL JEM‐ARM200F). The surface topography and roughness were measured by atomic force microscopy (AFM) using a Bruker Dimension Icon AFM in tapping mode.

### Electrochemical Characterization

The Pt powder coated on carbon cloth (denoted as Pt@CC) was used as the contrast sample. The platinum powder was the same raw material used above, and the loading amount of Pt@CC matches that of the Pt_1_Ga_420_ sample (≈6.67 mg). The ink of Pt@CC consists of 10 mg Pt powder, 965 µL isopropanol, and 35 µL Nafion solution (5 wt.%). Linear sweep voltammetry (LSV) curves were conducted in 1 M KOH at a scan rate of 10 mV s^−1^ to measure current density. And the electrochemical impedance spectroscopy (EIS) was measured in the frequency range of 10^5^ Hz–0.01 Hz with a 10 mA amplitude to evaluate interfacial charge‐transfer resistance (R_ct_) and electrolyte resistance (R_s_). The real‐time Pt segregation under applied potentials was observed via in situ optical microscopy. In situ ATR‐SEIRAS and Raman spectroscopy were measured in a 1 M KOH solution.

### In Situ Test Characterization

In situ Raman spectroscopy and ATR‐SEIRAS measurements under different potentials in the catalytic reaction environment were conducted by coupling the system with an electrochemical workstation. For the in situ Raman experiments, 0.3 g Pt@Ga catalyst was loaded onto a custom‐made gold electrode with a groove as the working electrode, while a saturated Ag/AgCl electrode and a Pt electrode were used as the reference and counter electrodes, respectively. The Raman spectra during the HER process were collected in the wavenumber range of 500 ~4000 cm^−1^ in 1 M KOH electrolyte. The laser power was set at 50%, with an exposure time of 10 s and 3 spectral accumulations. For the in situ ATR‐SEIRAS measurements, a VERTEX 70v Fourier transform infrared (FTIR) spectrometer equipped with a liquid‐nitrogen‐cooled MCT detector was employed. In this setup, 1 g Pt@Ga material was mixed with 30 mL of Nafion solution and subsequently dispersed in 1970 mL of anhydrous isopropanol under continuous N_2_ purging. After 20 min of ultrasonication, a gray, well‐dispersed ink solution was obtained. This ink was then uniformly loaded onto the surface of a Si‐based ATR prism and dried. The corresponding spectra were collected in a 1 M KOH solution. In the in situ optical microscopy tests, a homemade electrolytic cell was used for the measurements. In this setup, the liquid metal‐based catalyst was connected to a Cu wire, and a saturated Ag/AgCl electrode and a carbon electrode were used as the reference and counter electrodes, respectively. Under a 1 M KOH electrolyte, different potentials were applied to observe the surface morphological changes of the liquid metal‐based catalyst. The observation was carried out with a 50× objective lens.

## Conflict of Interest

The authors declare no conflict of interest.

## Supporting information



Supporting Information

## Data Availability

The data that support the findings of this study are available from the corresponding author upon reasonable request.
