# Supplementary material for: Dynamic Interfacial Modulation in Pt@Ga Liquid Metal Systems
Source: Adv Sci (Weinh). 2026 Jan 4;13(12):e16511. doi: 10.1002/advs.202516511 (PMC12948278; doi:10.1002/advs.202516511)
Supplement: Supplementary file 1 — Supporting Information [file ADVS-13-e16511-s001.docx]

Supporting Information

**Dynamic Interfacial Modulation in Pt@Ga Liquid Metal Electrocatalyst**

*Zanyu Chen, Yixiao Zou, Wenda Chen, Chen Zhang,* *Jinfeng Zhang, Jia Ding^*^, Xiaopeng Han**^*^ and Wenbin Hu^*^*

Z. Chen, Y. Zou, W. Chen, C. Zhang, J. Zhang, Prof. J. Ding, Prof. X. Han, and Prof. W. Hu
Tianjin Key Laboratory of Composite and Functional Materials, Key Laboratory of Advanced Ceramics and Machining Technology (Ministry of Education), School of Materials Science and Engineering, State Key Laboratory of Precious Metal Functional Materials, Tianjin University, Tianjin, 300350, China

Prof. X. Han, and Prof. W. Hu
National-Industry-Education-Platform of Energy Storage, Tianjin University, Tianjin, 300350, China

*Corresponding authors:

E-mail: jiading@tju.edu.cn (J. Ding); xphan@tju.edu.cn (X. Han); wbhu@tju.edu.cn (W. Hu)


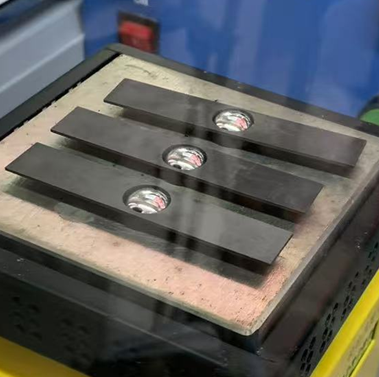


**Figure. S1** The preparation process of Pt@Ga materials.


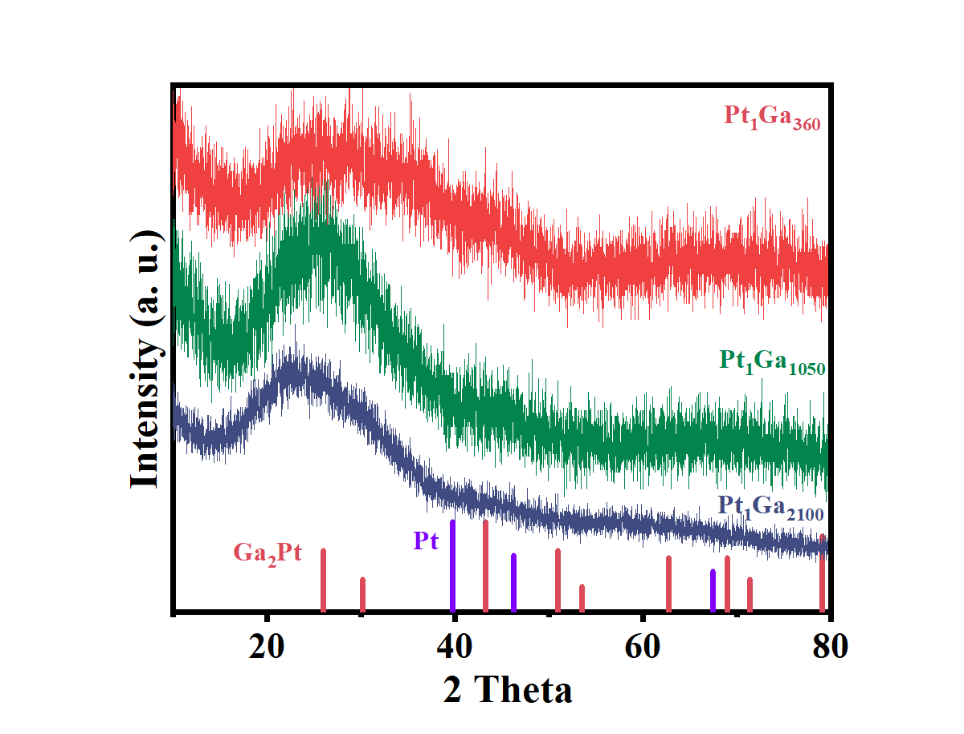


**Figure. S2** The XRD pattern of Pt_1_Ga_360_, Pt_1_Ga_1050_, Pt_1_Ga_2100_ materials.


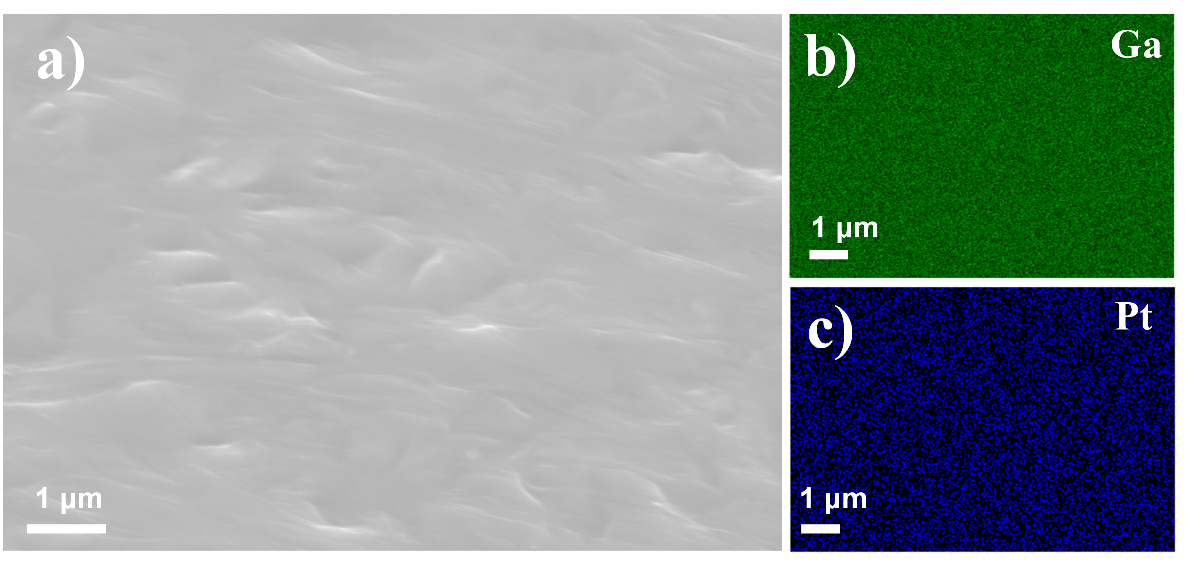


**Figure. S3** The SEM and mapping images the cooled Pt@Ga(Pt_1_Ga_420_) sample (without sonication) using environmental scanning electron microscopy (ESEM).


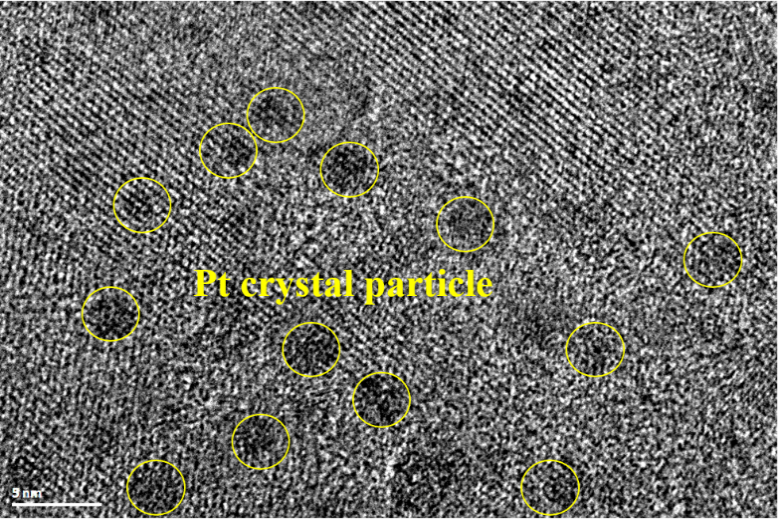


**Figure. S4** The HRTEM image of Pt_1_Ga_420_ (Pt@Ga) material.


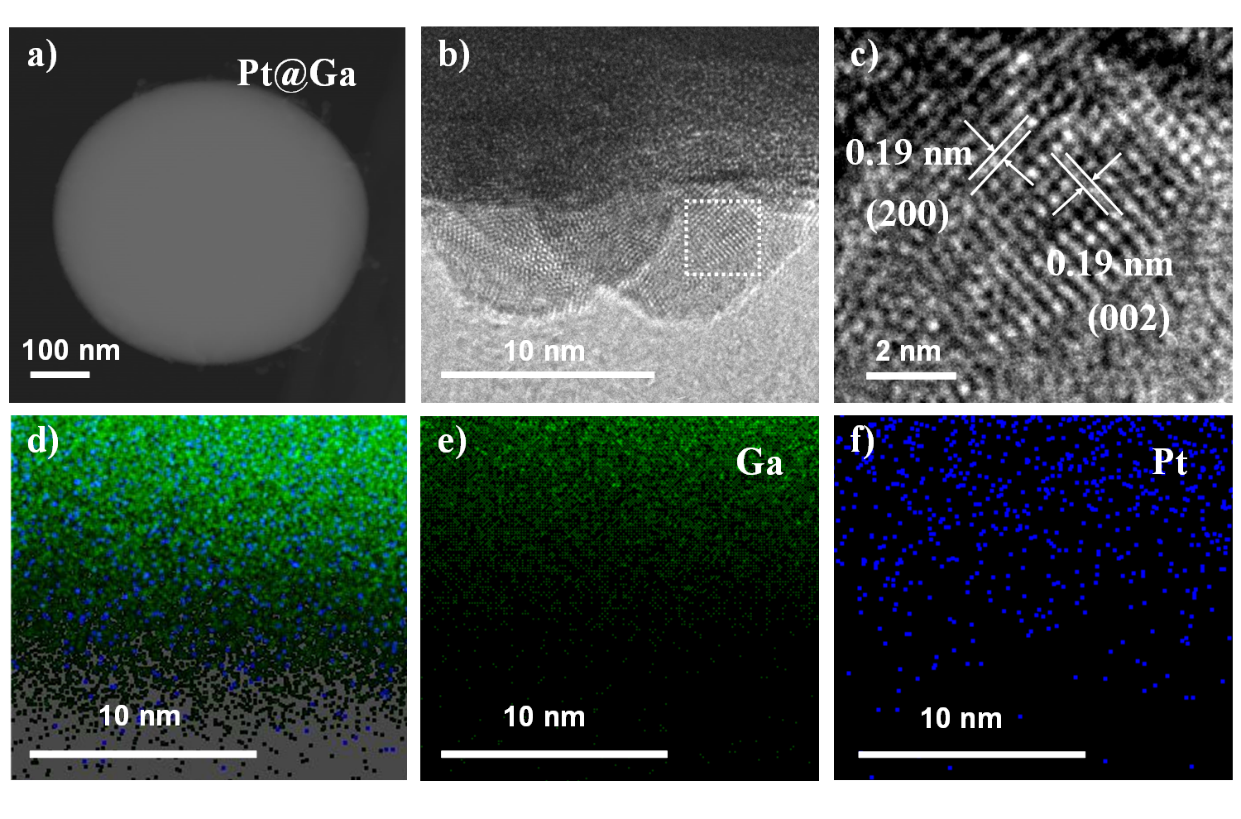


**Figure. S5** a) The HAADF image of Pt@Ga materials. b) and c) The high-resolution HRTEM images on the thin edge regions of the Pt@Ga droplets. d), e) and f) The EDX mapping of Pt@Ga materials.


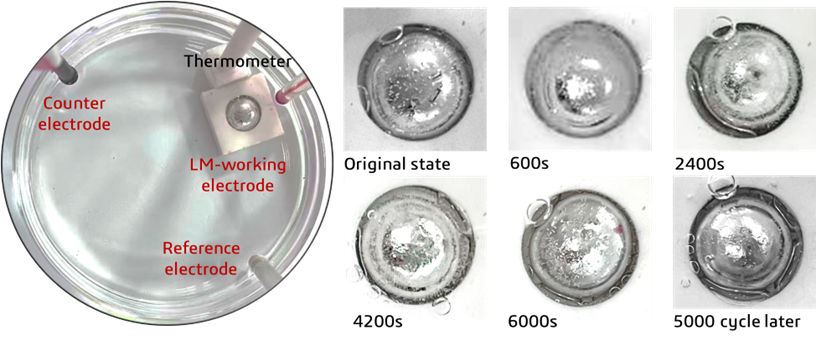


**Figure. S6** The self-made three-electrode system and the changes on the surface of the liquid metal during activation.


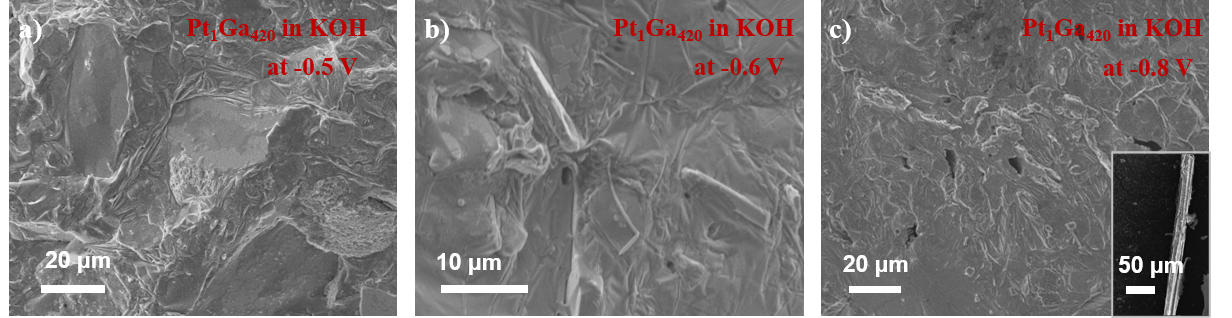


**Figure. S7** The SEM images of the Pt_1_Ga_420_ activated in KOH at the potential of a) -0.5 V, b) -0.6 V, and c) -0.8 V for 6000 s.


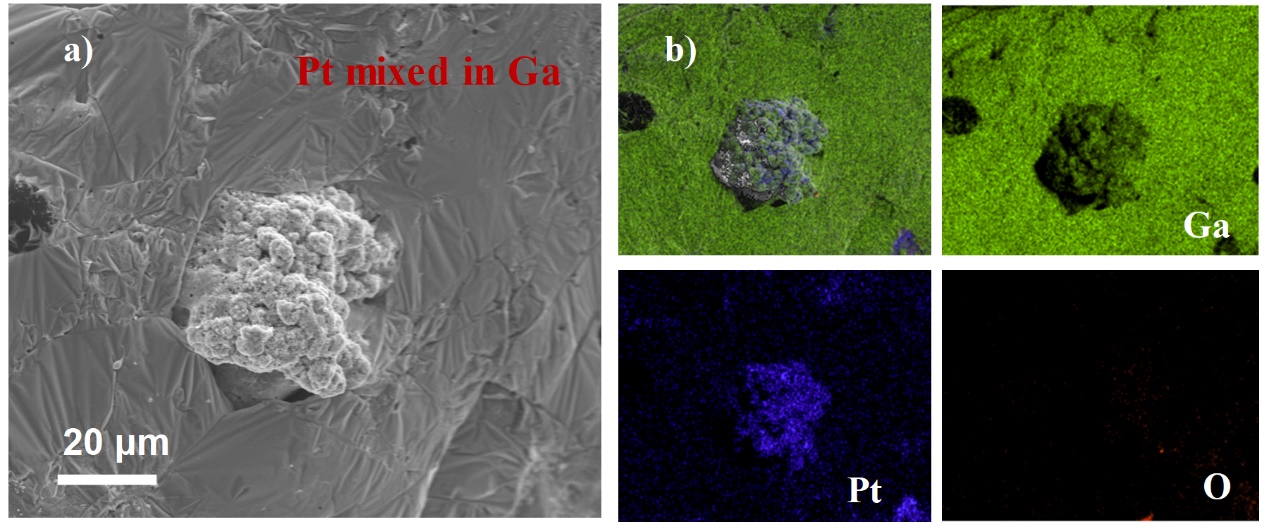


**Figure. S8** The a) SEM and b) EDX mapping images of Pt mixed in Ga.


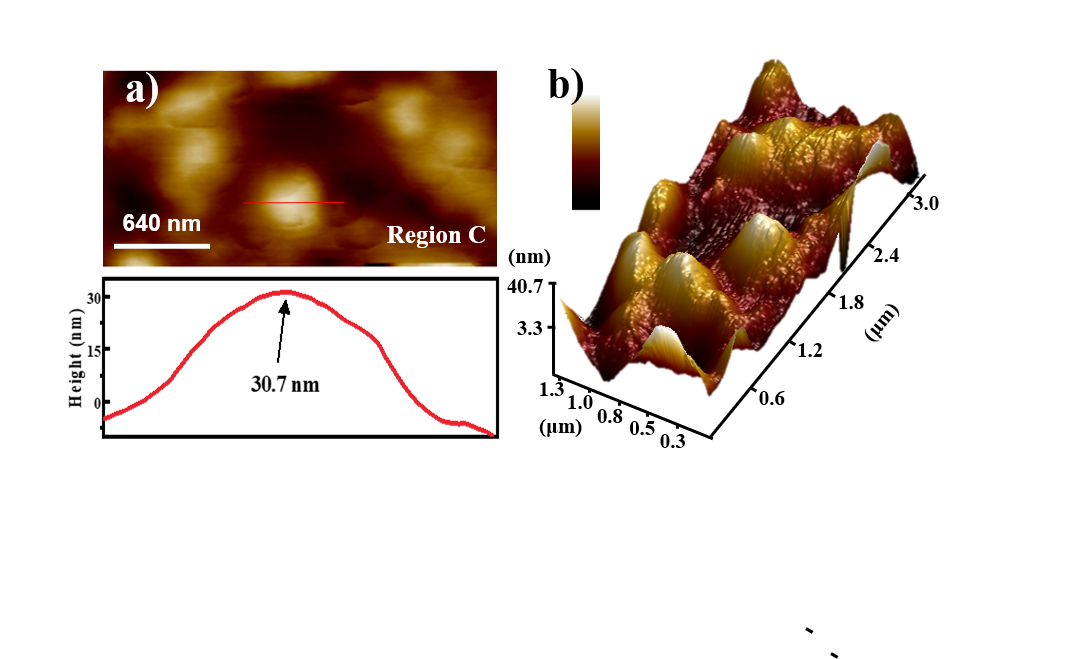


**Figure. S9** The AFM images of Pt@Ga-plate.


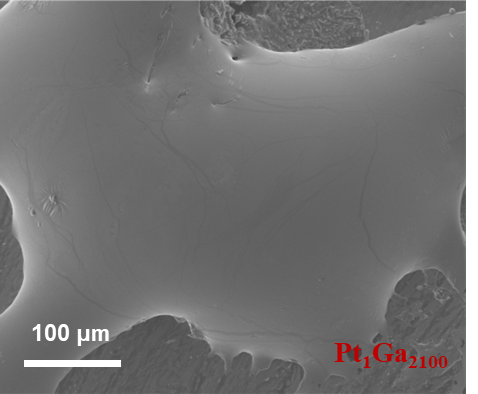


**Figure. S10** SEM image of Pt_1_Ga_2100_ after activating for 6000 s.


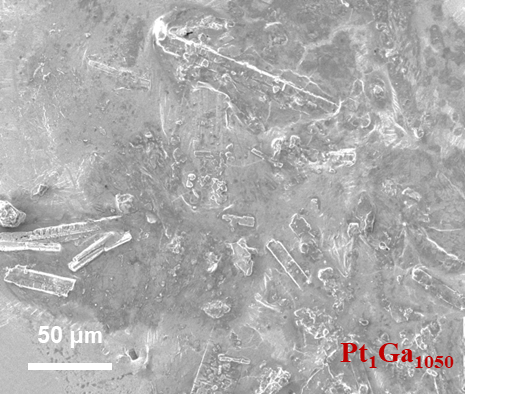


**Figure. S11** SEM image of Pt_1_Ga_1050_ after activating for 6000 s.


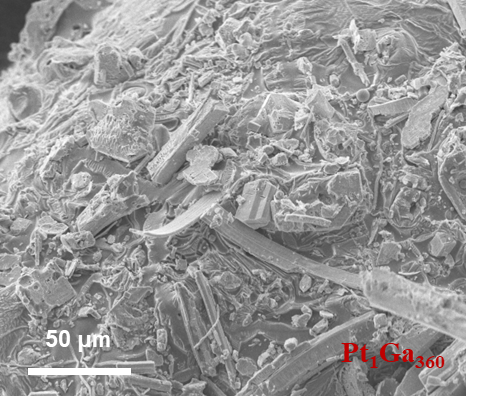


**Figure. S12** SEM image of Pt_1_Ga_360_ after activating for 6000 s.


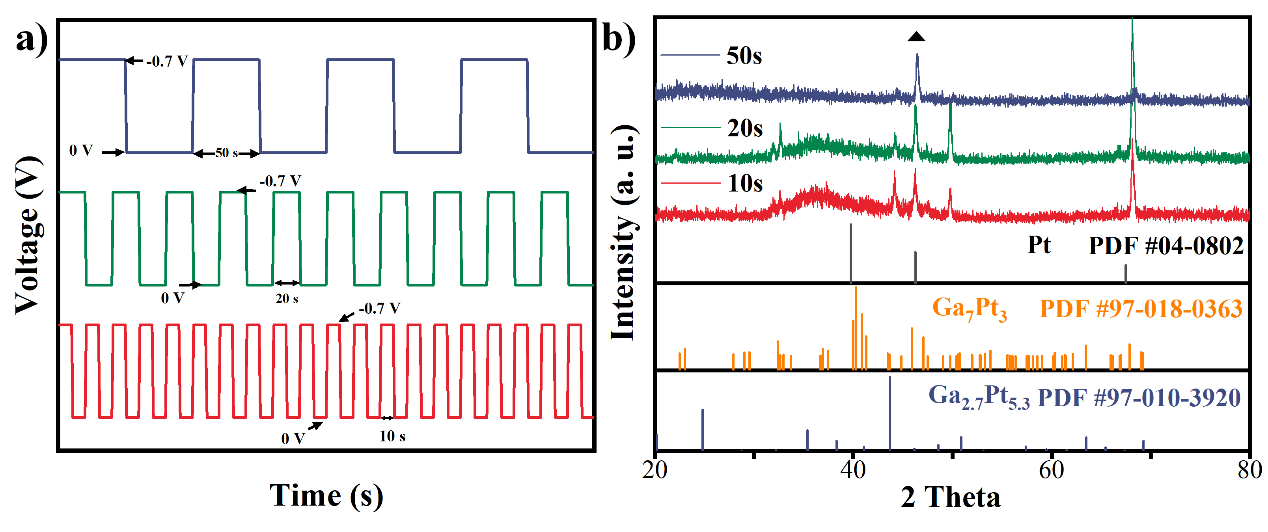


**Figure. S13** a) The voltage stimulation modes with intermittent protocols (50 s, 20 s, or 10 s intervals). b) The XRD pattern of the products.


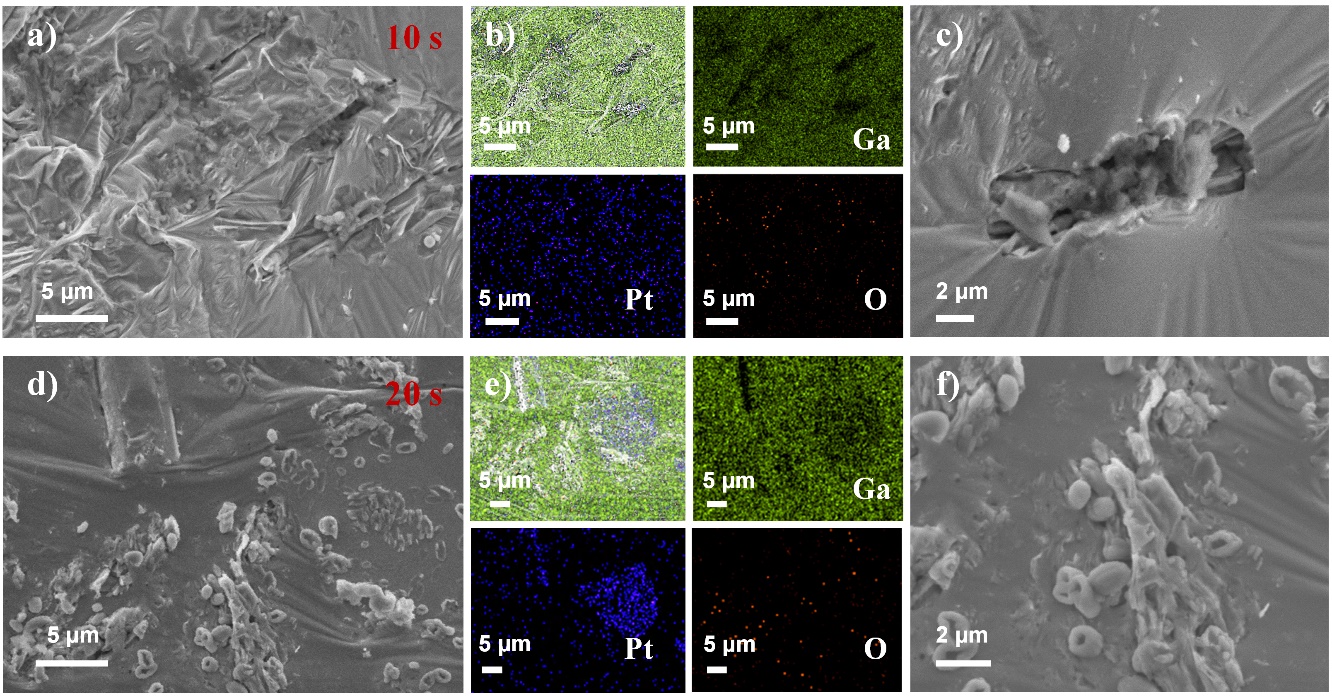


**Figure. S14** The SEM images and corresponding EDX-mapping characterization of liquid metal catalysts with voltage stimulation patterns: a-c) 10 s and d-f) 20 s.


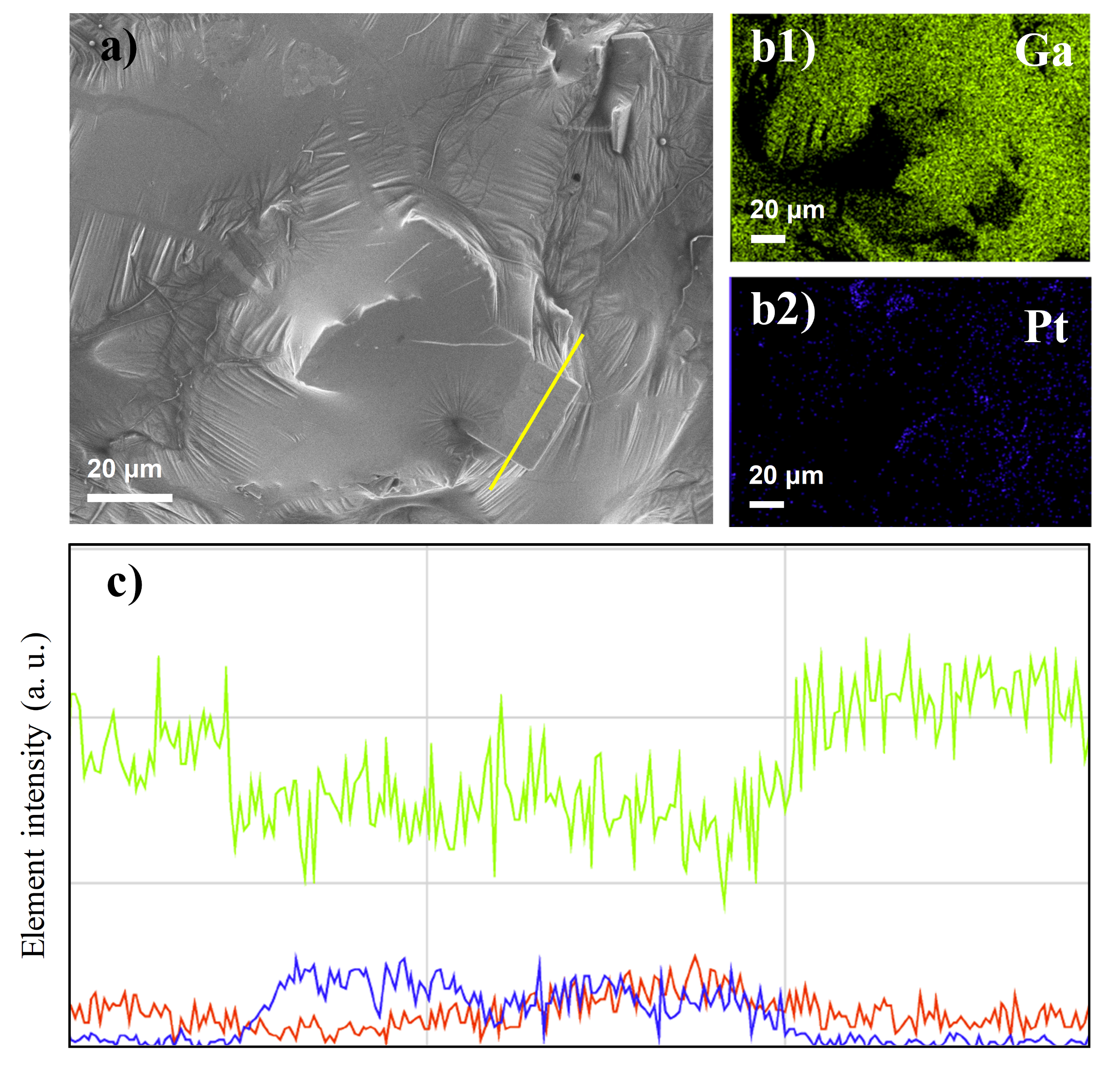


**Figure. S15** a) The SEM image, b) EDX mapping image and c) line-scan analyses of the Pt products with 50 s intervals.


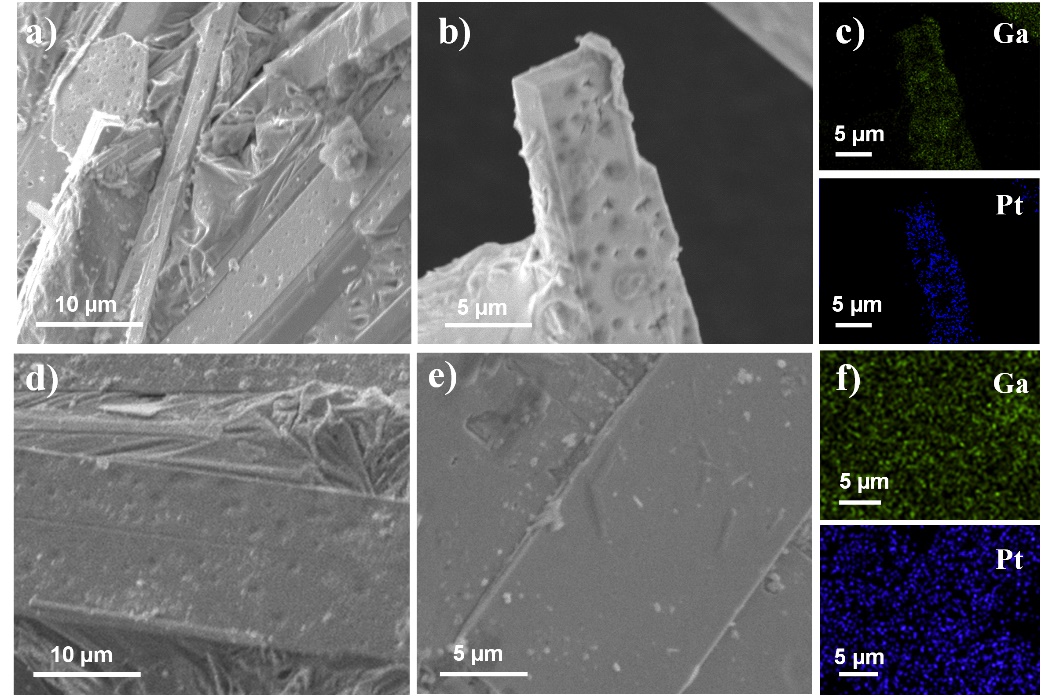


**Figure. S16** The SEM image and the correspond EDX-mapping image of catalysts’ surface in activation process: a-c) 1800 s and d-f) 4200 s.


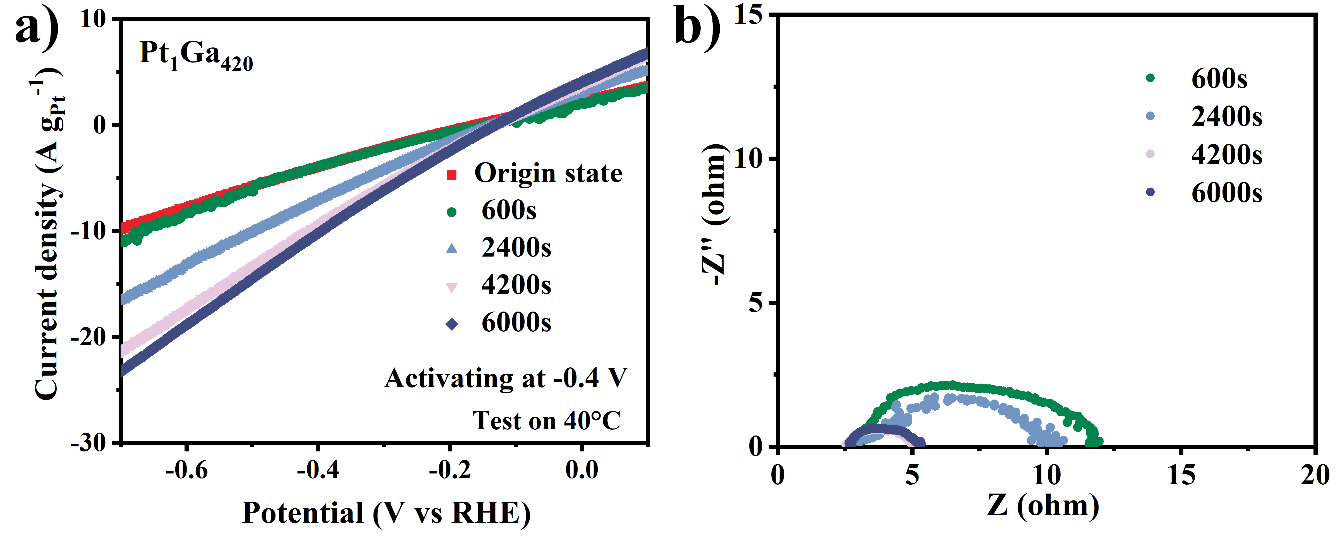


**Figure. S17** a) The HER activity and b) Nyquist plot of the Pt@Ga catalyst with electrochemical activation at -0.4 V.


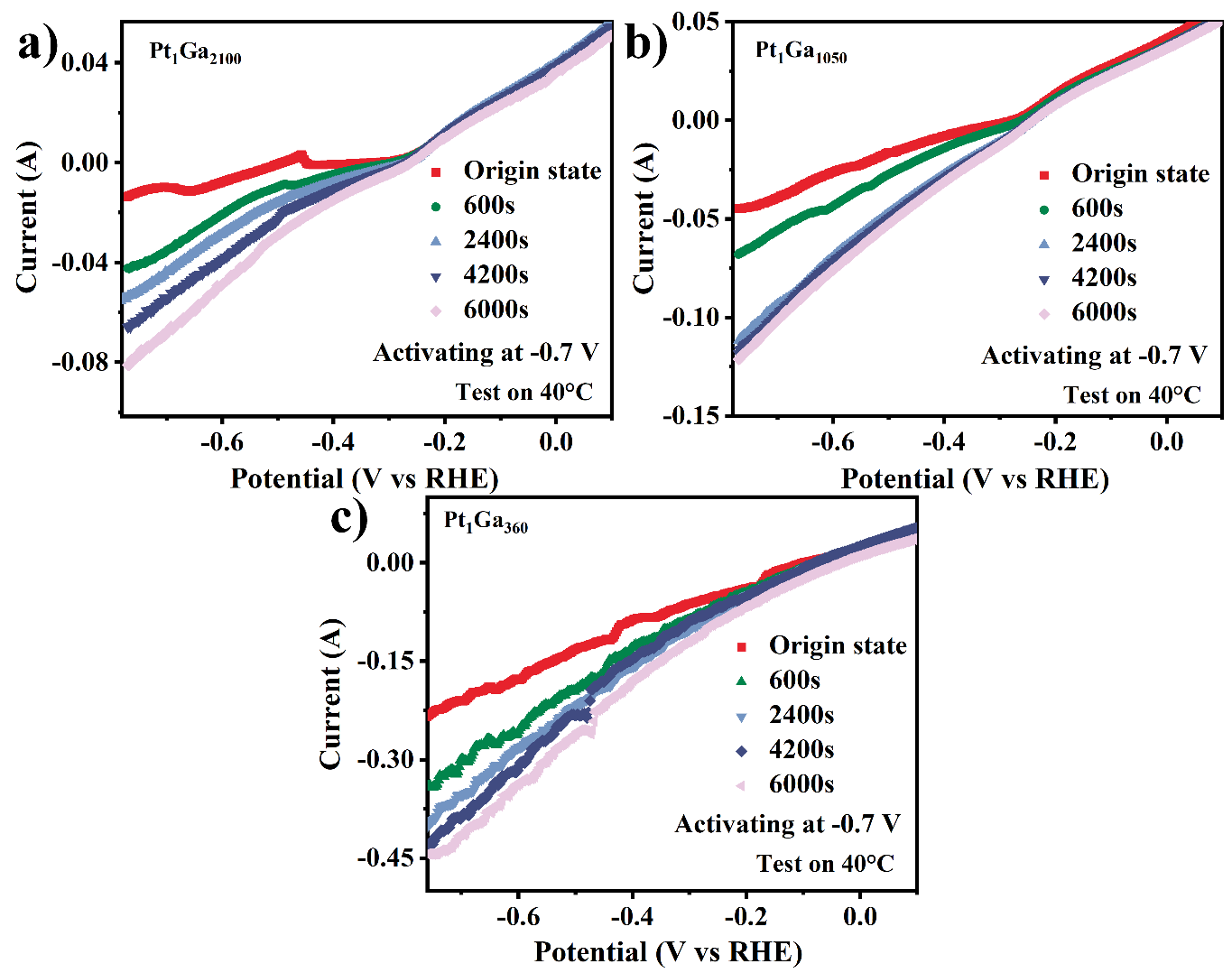


**Figure. S18** The LSV curves for HER region of Pt_1_Ga_2100_, Pt_1_Ga_1050_ and Pt_1_Ga_360_ samples.


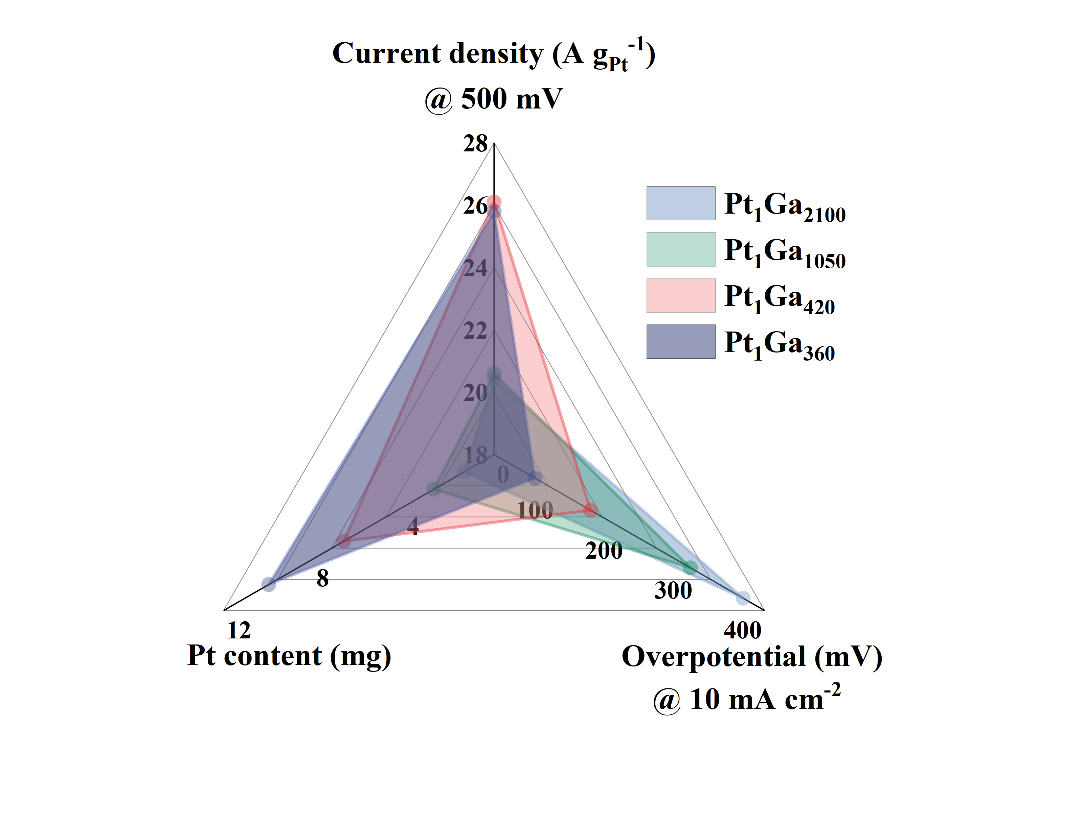


**Figure. S19** The comparison of Pt content, overpotential, current density of Pt_1_Ga_2100_, Pt_1_Ga_1050_, Pt_1_Ga_420_ and Pt_1_Ga_360_ samples.


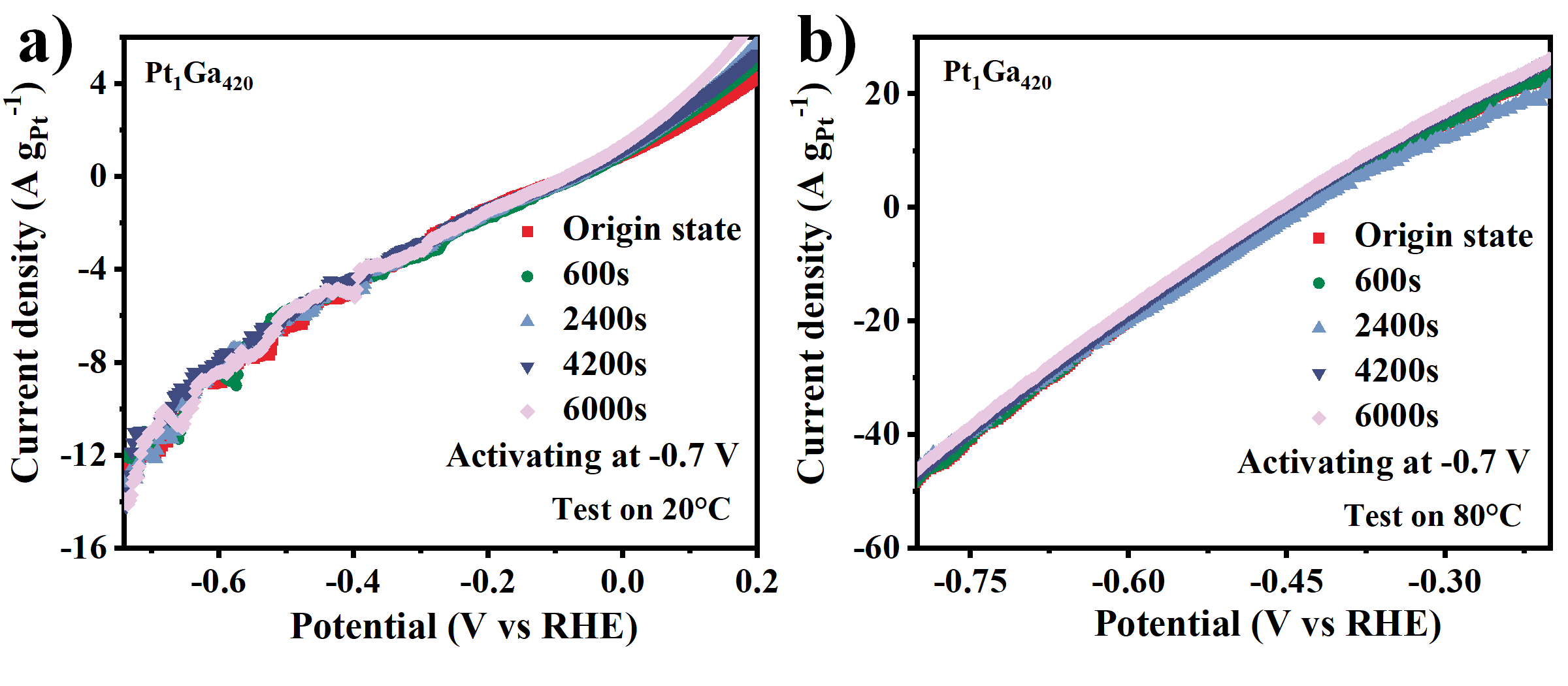


**Figure. S20** The Pt_1_Ga_420_ samples activating at -0.7 V test at a) 20°C and b) 80°C.


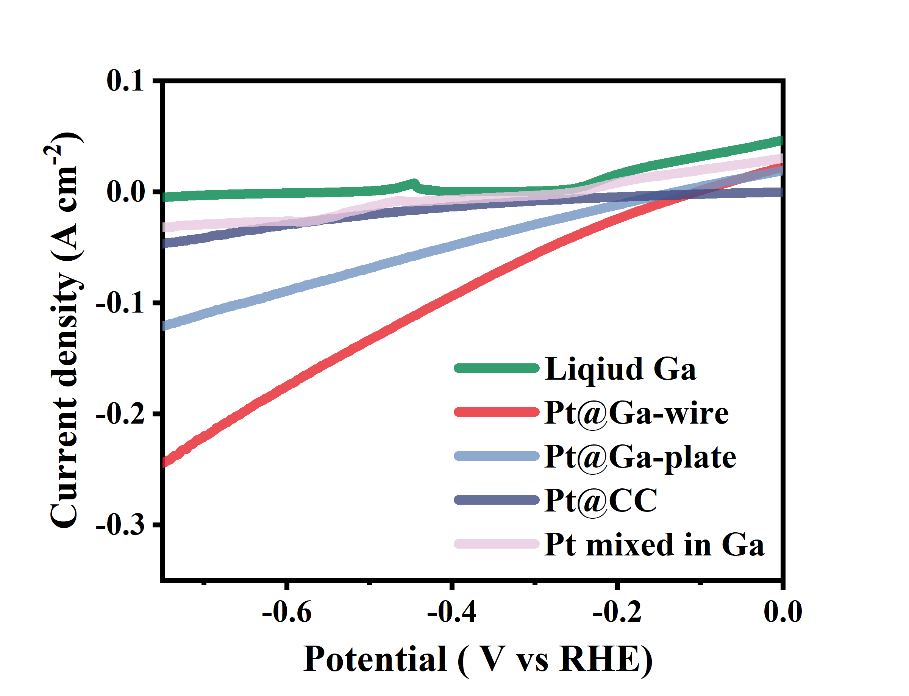


**Figure. S21** The HER performance of Liquid Ga, Pt@Ga-wire, Pt@Ga-plate, Pt mixed in Ga, and Pt@CC (calculated based on the area).


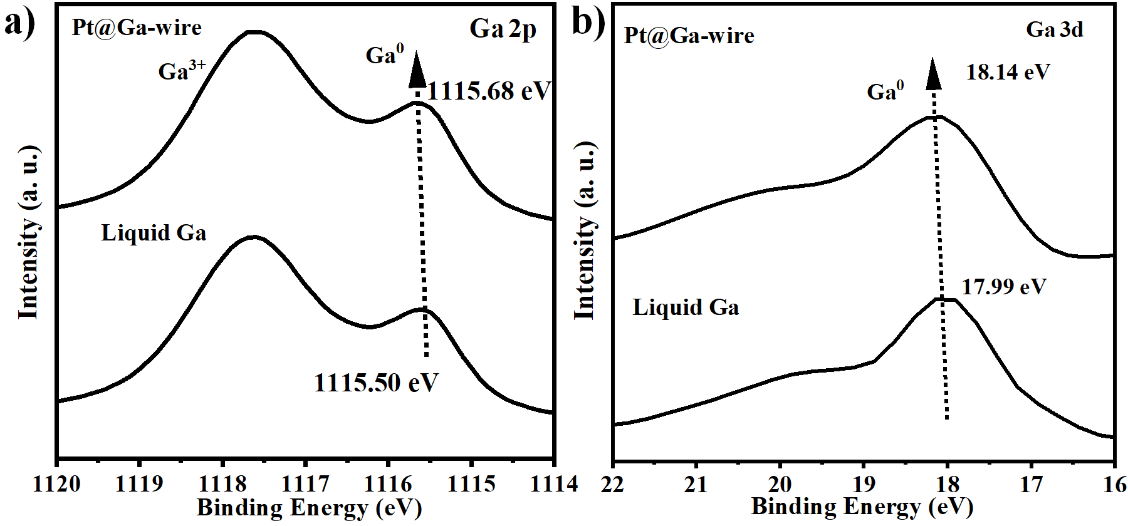


**Figure. S22** The high-resolution XPS spectra of (a) Ga 2p and (b) Ga 3d of Pt@Ga-wire and liquid Ga.


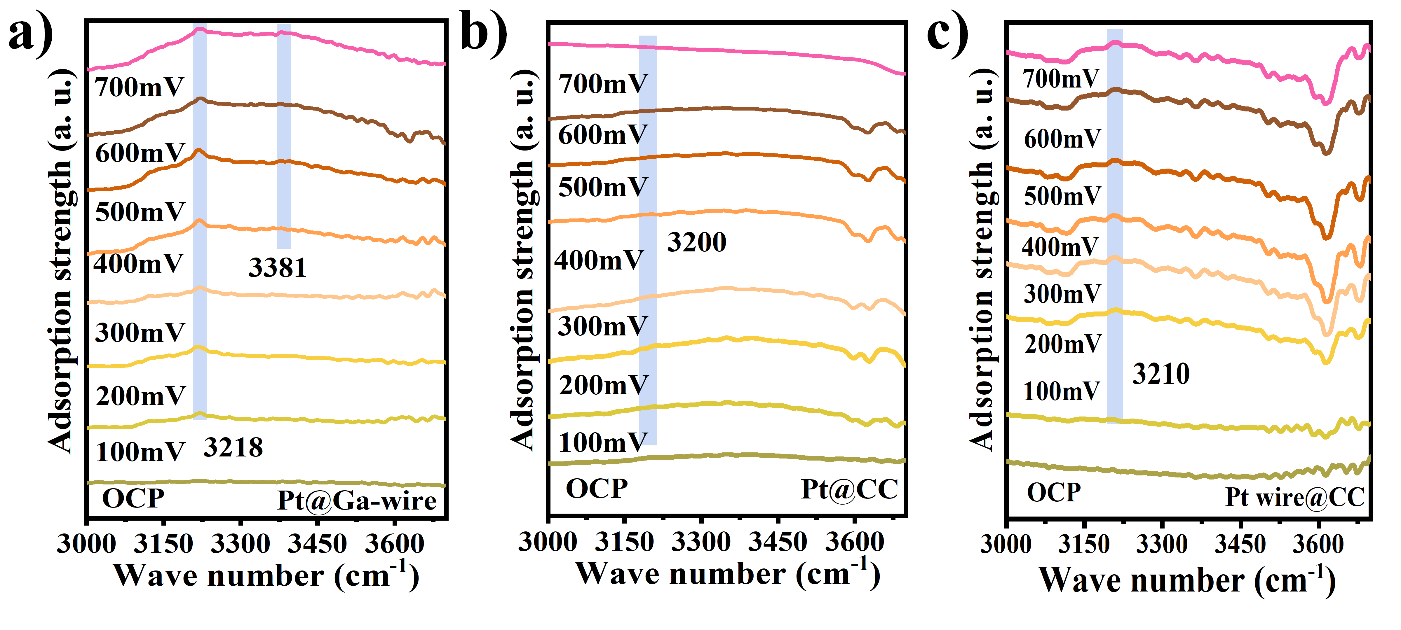


**Figure. S23** The characteristic OH vibrating peaks of a) Pt@Ga-wire, b) Pt@CC and c) Pt wire@CC measured via *in-situ* ATR-SEIRAS.


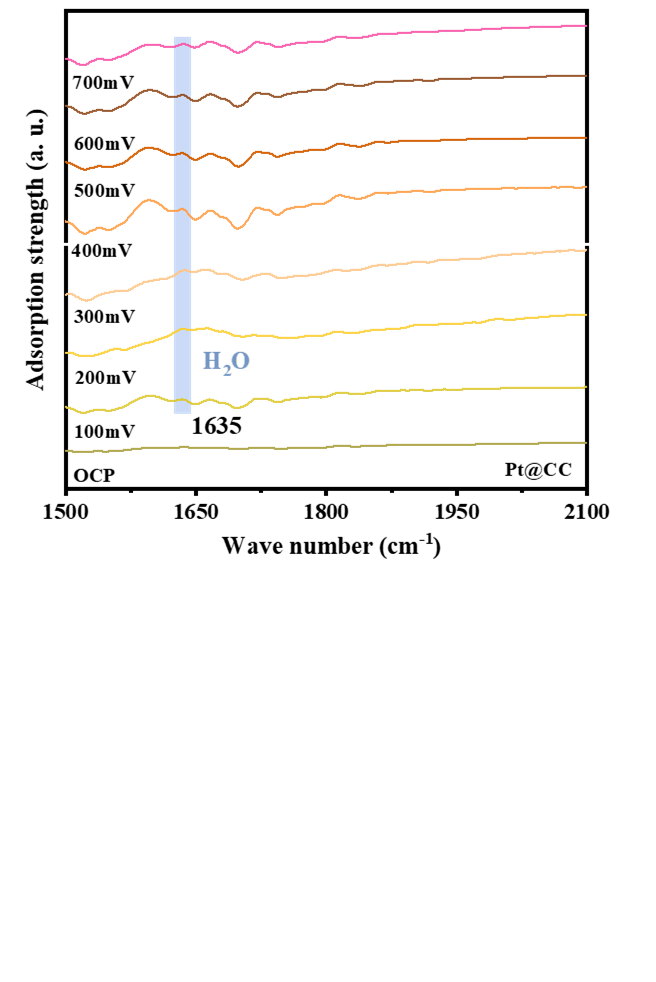


**Figure. S24** *In-situ* ATR-SEIRAS spectra of Pt@CC sample.

**
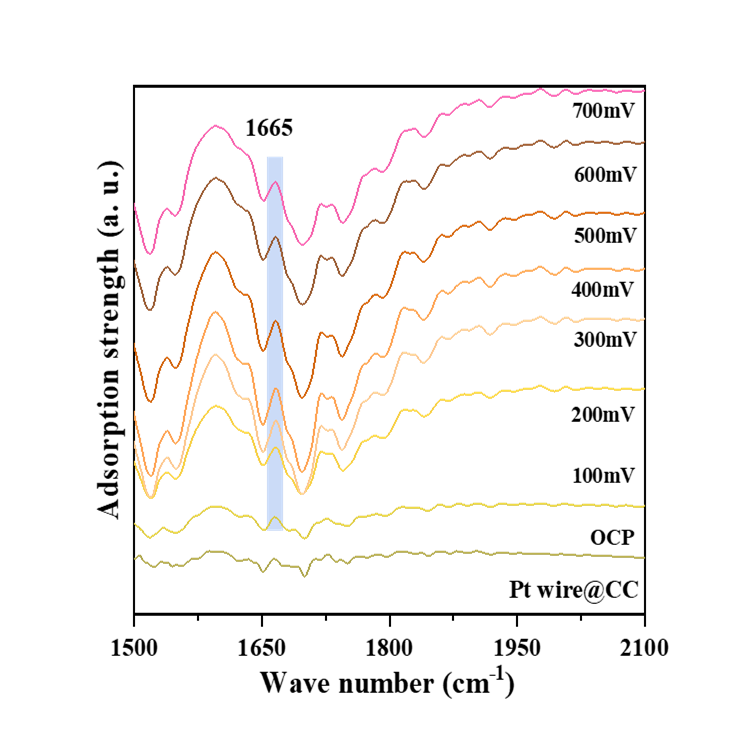
**

**Figure. S25** *In-situ* ATR-SEIRAS spectra of Pt wire@CC sample.


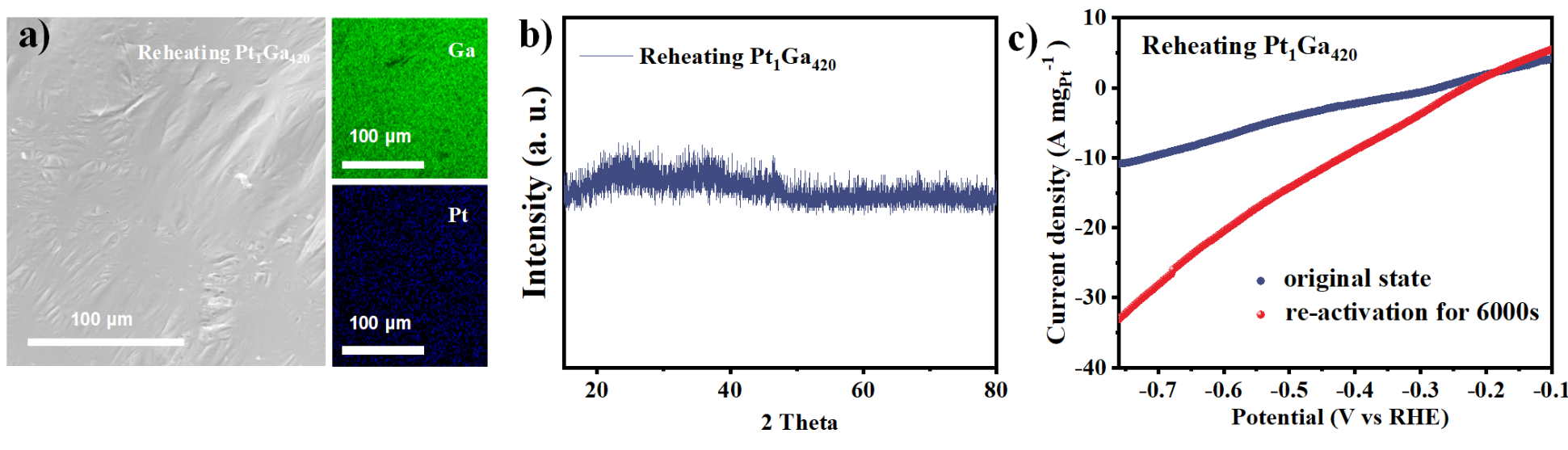


**Figure. S26** a) The SEM and the corresponding mapping images and b) the XRD pattern of reheating Pt_1_Ga_420_.


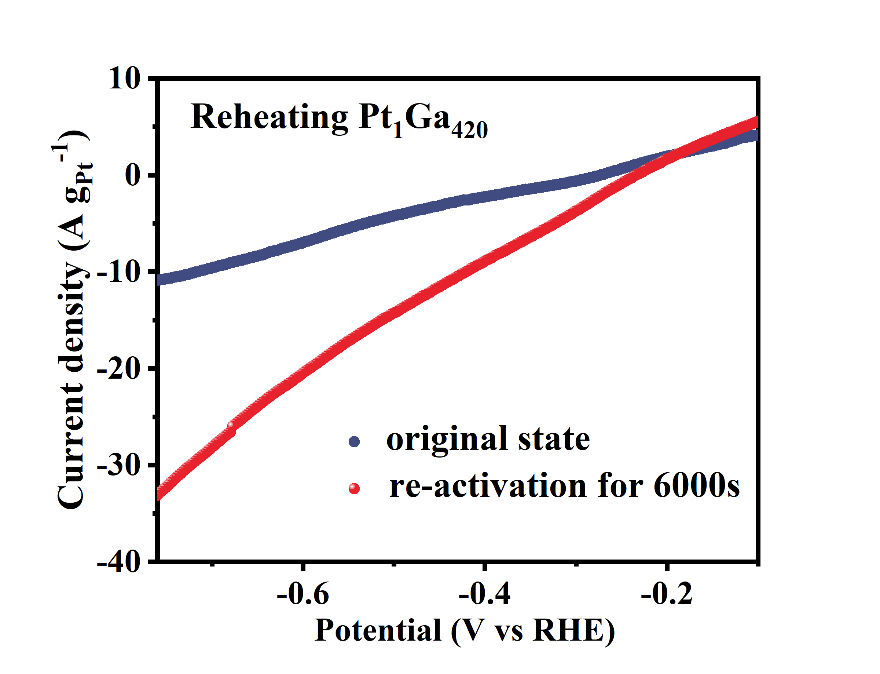


**Figure. S27** The LSV curves of reheating Pt_1_Ga_420_ before and after re-activation.

**Table S1** The fitted EIS results in Pt_1_Ga_420_.

| **State** | **R_s_** **(Ω)** | **R_ct_ (Ω)** |
| --- | --- | --- |
| Origin state | 2.5 | 10.1 |
| 600s | 1.65 | 7.77 |
| 2400s | 1.64 | 4.81 |
| 4200s | 1.64 | 4.37 |
| 6000s | 1.63 | 3.87 |

**Table S2** The fitted EIS results in different Pt-contained materials.

| **Samples** | **R_s_ (Ω)** | **R_ct_ (Ω)** |
| --- | --- | --- |
| Pt@Ga-wire | 1.63 | 3.87 |
| Pt@CC | 2.27 | 17.5 |
| Pt mixed in Ga | 1.72 | 19.9 |

**Table S3** The overpotential of the prepared samples.

| **Samples** | **Overpotential (mV)** |
| --- | --- |
| Pt@Ga-wire | 149@10 mA cm^-2^  284@50 mA cm^-2^ |
| Pt@Ga-plate | 191@10 mA cm^-2^  406@50 mA cm^-2^ |
| Pt mixed in Ga | 479@10 mA cm^-2^ |
| Pt@CC | 340@10 mA cm^-2^  770@50 mA cm^-2^ |

**Table S4** The element content of Ga in electrolyte with Pt@Ga catalyst.

| **Time** | **Content (mg L^-1^)** |
| --- | --- |
| 1800s | 31.50 |
| 6000s | 51.26 |
| 18000s | 51.86 |
| 36000s | 55.08 |
